# A novel cystatin derived from *Trichinella spiralis* suppresses macrophage-mediated inflammatory responses

**DOI:** 10.1371/journal.pntd.0008192

**Published:** 2020-04-01

**Authors:** Porntida Kobpornchai, Robin J. Flynn, Onrapak Reamtong, Nonglucksanawan Rittisoonthorn, Nathamon Kosoltanapiwat, Kobporn Boonnak, Usa Boonyuen, Sumate Ampawong, Montakan Jiratanh, Muncharee Tattiyapong, Poom Adisakwattana

**Affiliations:** 1 Department of Helminthology, Faculty of Tropical Medicine, Mahidol University, Bangkok, Thailand; 2 Department of Infection Biology, Institute of Infection and Global Health, University of Liverpool, Liverpool, United Kingdom; 3 Department of Molecular Tropical Medicine and Genetics, Faculty of Tropical Medicine, Mahidol University, Bangkok, Thailand; 4 Department of Microbiology and Immunology, Faculty of Tropical Medicine, Mahidol University, Bangkok, Thailand; 5 Department of Tropical Pathology, Faculty of Tropical Medicine, Mahidol University, Bangkok, Thailand; 6 National Institute of Animal Health, Department of Livestock Development, Ministry of Agriculture and Cooperative, Bangkok, Thailand; University Hospital Bonn, GERMANY

## Abstract

*Trichinella spiralis* can modulate host immune responses to retain a suitable environment for its long-term survival. Incidentally, the parasite elicits regulatory effects through immunomodulatory molecule release, which can suppress host inflammation and may be used for the treatment of unrelated inflammatory diseases in someday. Here we identified and characterized a novel *T*. *spiralis* cystatin (TsCstN), which inhibits inflammation mediated by LPS-treated macrophages.Proteins contained in the excretory–secretory (ES) product of muscle-stage *T*. *spiralis* (ES-L1) were fractionated, and each was treated with mouse bone marrow-derived macrophages (mBMDMs) before LPS stimulation. The fractions that exhibited high immunomodulatory property by decreasing pro-inflammatory cytokines or increasing anti-inflammatory cytokines were identified by mass spectrometry. Incidentally, the conserved hypothetical protein (Tsp_04814) was selected for further characterization as it presented the most significant MS score. An annotation of Tsp_04814 using protein structural homology comparison suggested that it has high structural similarity to human cystatin E/M (TM score 0.690). The recombinant *T*. *spiralis* novel cystatin (rTsCstN) was expressed in *Escherichia coli* at a molecular weight of approximately 13 kDa. Mouse anti-rTsCstN polyclonal antibody (pAb) could detect native TsCstN in crude worm antigens (CWA) and ES-L1 and be predominantly localized in the stichosome and subcuticular cells. rTsCstN inhibited cysteine proteases *in vitro*, especially cathepsin L, at an optimal pH of 6. Besides, rTsCstN could be internalized into mBMDMs, which were mostly distributed in the cytoplasm and lysosome both before and after LPS stimulation. To evaluate the rTsCstN immunomodulatory properties on mBMDMs, rTsCstN was incubated with mBMDM before LPS stimulation; this demonstrated that rTsCstN suppressed pro-inflammatory cytokine production and MHC class II expression.*T*. *spiralis* L1-derived TsCstN was characterized as a novel cysteine protease inhibitor. The protein elicits an anti-inflammatory property by suppressing pro-inflammatory cytokines and interfering with the antigen presentation process through depletion of MHC class II expression.

## Author summary

During infection, *T*. *spiralis* secretes ES products to modulate host immune responses by suppressing inflammation and stimulating immunoregulatory cells, especially macrophages, which are key in both innate and adaptive immune responses. Here, we attempt to identify a novel ES-L1-derived immunomodulatory molecule, which can suppress inflammation of LPS-stimulated mBMDMs and promote alternatively activated macrophages (M2). TsCstN, a novel cystatin identified in ES fractions of ES-L1, exhibits immunomodulatory properties on mBMDMs. rTsCstN demonstrated cysteine protease inhibition, specifically to cathepsin L (CatL). The immunomodulatory property of rTsCstN was evaluated by the treatment of LPS-stimulated mBMDM and demonstrated that rTsCstN could suppress inflammation and interfere with antigen processing by downregulating pro-inflammatory cytokines and MHC class II expression (MHC-II) of mBMDMs, respectively. Our finding provides the initiative for explaining the molecular basis of how TsCstN modulates host immune responses, which might be useful for the future development of an alternative treatment against inflammatory diseases.

## Introduction

*Trichinella spiralis*, a causative agent of trichinellosis, is a harmful foodborne zoonotic nematode that infects a variety of mammalian hosts, including humans [1]. After consumption of raw or poorly cooked infected meat, the muscle larvae (L1) emerge and migrate to the small intestine where they develop into an adult stage and subsequently release newborn larvae (NBL), which migrate into tissues and directly penetrate striated muscle cells [2]. To survive in their host, the parasites utilize immune evasion mechanisms, such as anatomical seclusion, and this includes intracellular infection and nurse cell formation [3] and regulation of host immune responses by releasing immunomodulatory molecules present in their excretory–secretory (ES) products [4].

Macrophages are important effector cells that respond during the *T*. *spiralis* infection [5]. However, there is ample evidence to indicate that macrophage functions are regulated by *T*. *spiralis* ES products (TsES) [5]. Treatment of J774A.1 or RAW264.7 macrophage cell lines with TsES significantly reduced the expression of LPS-induced pro-inflammatory cytokines, TNF-α, IL-1β, IL-6, and IL-12 but increased the expression of regulatory cytokine; this effect was observed even in the presence of ES products alone [5, 6]. Adoptive transfer of macrophages obtained from *T*. *spiralis*-infected mice or TsES-treated macrophages into colitis or airway hyperresponsiveness (AHR) mouse models could ameliorate disease severity [7]. Although there have been several studies demonstrating TsES immunomodulatory capacity, few have focused on the characterization of effective molecules, and many of these molecules need to be further identified. A recombinant *T*. *spiralis-*specific 53 kDa glycoprotein (rTsP53) obtained from TsES prevented trinitrobenzene sulfonic acid (TNBS)-induced colitis and LPS-induced lethal sepsis in mice through the induction of alternatively activated or M2 macrophages; the decrease of pro-inflammatory cytokines, IL-6, IFN-γ, and TNF-α; and the increase of regulatory cytokines, IL-10 and TGF-β [8, 9]. Furthermore, the administration of a recombinant *T*. *spiralis* cathepsin B-like protein (rTsCPB) ameliorated intestinal ischemia/reperfusion (I/R) injury in mouse models *via* the induction of M2 macrophages [10]. These studies suggest that individual molecules can be identified and produced to recapitulate the beneficial effects of native TsES.

As mentioned above, we aimed to identify and functionally characterize novel proteins responsible for driving a regulatory phenotype in mouse bone marrow-derived macrophages (mBMDMs). TsES-L1 was fractioned using anion exchange chromatography and subsequently used to treat LPS-stimulated mBMDM. Cytokine profiles were monitored by qRT-PCR to determine fractions with the greatest immunomodulatory capacity. Four fractions, which elicited high immunomodulatory effects, were subjected to protein identification using nano-LC/MS/MS. Following protein identification, we successfully cloned and expressed a novel *T*. *spiralis* cystatin (TsCstN), a 13.2 kDa protein with low amino acid sequence conservation but high structural homology to other cystatin homologs. Herein, we further characterized the basic properties and functions of this novel cystatin isoform, including its immunomodulatory capacity.

## Materials and methods

### Animal and ethics statement

Six to eight-week-old female ICR mice were purchased from the National Laboratory Animal Center, Mahidol University, Salaya Campus, Nakhon Pathom, Thailand, and 4–6-week-old female BALB/c mice from the Nomura Siam International Company, Bangkok, Thailand. The mice were housed in the Animal Care Unit, Faculty of Tropical Medicine, Mahidol University, Bangkok, Thailand. In this study, housing and all experiments were performed on mice according to the Ethical Principles and Guidelines for the Use of Animals of the National Research Council of Thailand and with permission from the Faculty of Tropical Medicine Animal Care and Use Committee (FTM-ACUC), Mahidol University, with approval number FTM-ACUC025/2017.

### Preparation of excretory–secretory products and fractionation

Muscle-stage larvae (L1) of *T*. *spiralis* were prepared by standard pepsin digestion (0.8% w/v Pepsin, 0.8% v/v HCl) to release larvae from the striated muscles of ICR mice, as previously described [11]. Approximately 200 *T*. *spiralis* L1s were used to orally infect 6- to 8-week-old female ICR mice, and the infected mice were sacrificed at 35 days post-infection (dpi). The *T*. *spiralis* L1 was obtained as previously described [11]. Excretory–secretory (ES) products of L1 (ES-L1) were prepared as described previously with some modifications [12]. In summary, L1 obtained from mice were immediately cultured in RPMI-1640 medium (GE Healthcare Life Sciences, South Logan, UT) supplemented with 1 × penicillin/streptomycin (Biowest SAS, Nuaillé, France) in 5% CO_2_ at 37°C for 18 h followed by dialysis and concentration using Amicon Stirred cell (EMD Millipore Corporation, Billerica, MA) through an ultracel 3 kDa ultrafiltration disk (EMD Millipore). ES was sterilized by filtering through a 0.2 μm Nalgene syringe filter (Thermo Scientific Nalgene Product, Rochester, NY) before storage at –80°C until use.

The level of ES proteins was determined by the Coomassie Plus Protein Assay (Thermo Fisher Scientific Inc., Waltham, MA) according to the manufacturer’s instructions before anion exchange chromatography fractionation as previously described [13]. Briefly, the ES products were dialyzed against 20 mM Tris-HCl, pH 8.6 for 16–18 h, at 4°C. Subsequently, the protein solution was loaded into an anion exchange chromatography column (HiTrap Q HP, GE Healthcare) and washed several times with 20 mM Tris-HCl, pH 8.6. The proteins were eluted by NaCl gradient in 20 mM Tris-HCl, pH 8.6, followed by analyzing on SDS-PAGE and silver staining. Each protein fraction was dialyzed against 1 × PBS and then sterilized by filtering through a 0.2 μm Nalgene syringe filter (Thermo Scientific Nalgene Product) before storage at –80°C until use.

### Culture of murine bone marrow-derived macrophages

The mouse bone marrow-derived macrophages (mBMDMs) were prepared from BALB/c mice according to the Bowdish Lab protocol [14]. Briefly, the bone marrow cells were isolated by cutting both ends of the femur and tibia, followed by flushing out using a needle attached to a syringe containing RPMI-1640 (GE Healthcare). The cells were cultured in 15 ml of macrophage complete medium [RPMI-1640 (GE Healthcare) containing 10% FBS (Biowest), 2.5 mM HEPES, 1 × penicillin/streptomycin (Biowest), and 20% L-929 conditioned medium (LCM)] and incubated in 5% CO_2_ at 37°C for 7 days. Three days later, a fresh macrophage complete medium was added, and incubation continued for another 3 days. After incubation, non-adherent cells were removed before adding 25 ml of fresh macrophage complete medium and then incubating overnight. On the following day, the adherent cells were detached by adding 2 mM EDTA followed by scrapping using a cell scraper. Cell viability was determined by the trypan blue exclusion technique [15], and the purity of mBMDMs was determined by the MACSQuant flow cytometer (Miltenyi Biotec), which presented 70%–80% of F4/80^+^ and CD11b^+^ cells.

### Stimulation of mBMDMs

The mBMDMs were plated into 24-well culture plates at a density of 2 × 10^5^ cells per well and then incubated at 37°C with 5% CO_2_ for 24 h. Treatment of mBMDM was conducted in two ways: 1) The cells were treated with 10 μg/ml of each ES fraction and subsequently incubated at 37°C with 5% CO_2_ for 24 h before stimulating with 100 ng/ml of LPS (*E*. *coli* 0111: B4) for 12 h. 2) The mBMDM cells were treated with 10 μg/ml of recombinant *T*. *spiralis* novel cystatin (rTsCstN) or 10 μg/ml of recombinant mouse dihydrofolate reductase (rmDHFR) (an irrelevant control) at 37°C with 5% CO_2_ for 24 h, followed by stimulating with LPS (*E*. *coli* 0111: B4) and additionally incubating for 12 and 24 h, respectively. Three different control groups were included in all experiments, comprising treatment with only recombinant protein or ES fraction, treatment with only LPS, and cell culture media (untreated control). After incubation, the culture media and the cells were collected to monitor cytokine levels and mRNA expression using ELISA and quantitative real-time RT-PCR (qRT-PCR), respectively. The experiment was performed in triplicates with two independent experiments.

### Determination of cytokine levels

The cells were harvested by adding TRIZOL reagent (Invitrogen) according to the manufacturer’s instructions, and total RNA was subsequently isolated. Genomic DNA contamination was removed by treating with DNaseI (Thermo Fisher Scientific). After that, the DNA-free total RNA was reverse-transcribed by the RevertAid First strand cDNA Synthesis Kit (Thermo Fisher Scientific). The transcription levels of cytokine genes and macrophage phenotype markers were determined using qRT-PCR (Bio-Rad Laboratories, Inc., Hercules, CA). The specific primers were designed as listed in S1 Table. Amplification was performed using the MasterCycler Real-Time PCR system (Realplex4, Eppendorf, Hamburg, Germany), and it consisted of a first denaturation step at 95°C for 5 min, followed by 40 cycles at 95°C for 15 s and 60°C for 60 s and a melting curve step at 65°C–95°C. Relative mRNA expression was calculated using the comparative Ct method with the formula 2^−ΔΔCt^ [16].

The levels of cytokines secreted from mBMDMs, including TNF-α, IFN-γ, TGF-β, IL-10, and IL-4, were determined in the culture supernatant using commercial sandwich ELISA kits according to the manufacturer’s instructions (eBioscience, Thermo Fisher Scientific). The cytokine concentrations were determined from the average of triplicates, and standard curves were generated with recombinant cytokines.

### Analysis of macrophage surface molecules

After the treatment of mBMDMs as described above, cell surface expression on mBMDMs was performed *via* flow cytometry [17]. The macrophages were blocked with blocking buffer (PBS with 10% FBS) on ice for 30 min and then stained with the monoclonal antibodies; APC anti-mouse CD80, FITC anti-mouse CD86, and PE anti-mouse MHC class II (IA/IE)) (eBiosciences, San Diego, CA). Isotype-matched mAbs were used for control staining. After washing with a cell staining buffer (PBS with 3%FBS), the cells were analyzed by the MACSQuant flow cytometer (Miltenyi Biotec).

### Mass spectrometry

Protein fractions that modulated cytokine responses of macrophages were subjected to mass spectrometry to identify their composition as described previously [18]. In summary, the protein samples were reduced and alkylated to free cysteine residues by incubating with dithiothreitol (DTT) and iodoacetamide (IAA), respectively, followed by digesting with trypsin. The peptides were extracted by acetonitrile and then dried using a vacuum evaporator. The protein pellets were reconstituted in formic acid and then analyzed by an Ultimate 3000 Nano-LC systems (Thermo Fisher Scientific) coupled with a microTOF-Q II (Bruker, Germany). The mass spectra were processed using Data Analysis TM 4.0 analysis software (Bruker and Mascot version 2.4.1, Matrix Science, London, UK)

### Sequence and bioinformatics analysis

In previous studies, the annotation of hypothetical proteins (proteins with unknown functions) was successfully relied on to predict protein structure and function [19, 20]. Therefore, the putative function of the conserved hypothetical protein (Ts_04814) (accession no. XP_003369399.1) was predicted using the I-TASSER protocol [21]. The results suggested that the conserved hypothetical protein (Ts_04814) has structural homology with human cystatin E/M (PDB ID. 4n6oB), and thus, it was renamed to *T*. *spiralis* novel cystatin (TsCstN). The nucleotide and amino acid sequences of TsCstN were obtained from the NCBI database (https://www.ncbi.nlm.nih.gov). The basic properties of the protein were evaluated using bioinformatics techniques, including an analysis of the molecular weight and isoelectric point using ProtParam tool on the ExPASy server [22], a prediction of the signal peptides (classical secretory pathway) using SignalP 4.1 [23], non-classical secretory pathway using SecretomeP 2.0 [24], transmembrane helix using TMHMM [25], and N- and O-linked glycosylation sites using NetNGlyc 1.0 [26] and NetOGlyc 4.0 [27]. Potential disulfide bond formation was predicted using DiANNA 1.1 web server [28]. Multiple sequence alignments of TsCstN and its homologs were performed using Clustal Omega [29].

### Cloning, expression, and purification of recombinant TsCstN protein

Total RNA was extracted from L1 using TRIZOL (Invitrogen, Carlsbad, CA) and then treated with DNase I (Thermo Fisher Scientific) before converting to first strand cDNA using the RevertAid First strand cDNA Synthesis Kit (Thermo Fisher Scientific). TsCstN cDNA without a signal peptide was amplified by PCR in a total volume of 20 μl containing 2 μl of first strand cDNA, 1 × *Taq* DNA polymerase buffer, 0.2 mM of each dNTP, 2 mM MgCl_2_, 1 U *Taq* DNA polymerases, and 100 nM specific primers (Fw: TAGGATCCGGATTTGAGTGAATTGGATGAAG and Rv: TAAAGCTTGACTGCATAATATGTGCATTC), where the *Bam*H I and *Hind* III restriction enzyme sites are underlined, respectively. The PCR conditions consisted of an initial step, comprising of 94°C for 5 min, 35 cycles of 94°C for 30 s, 56°C for 30 s, and 72°C for 5 min, and final extension step at 72°C for 5 min. PCR product was inserted into the pET-23b^+^ expression vector (Novagen-EMD Biosciences, Inc., Darmstadt, Germany) and transformed into an *E*. *coli* strain BL21 (DE3) (Novagen). Expression of recombinant TsCstN (rTsCstN) was induced by isopropyl-β-D-thiogalactopyranoside (IPTG; Thermo Fisher Scientific) at a final concentration of 0.2 mM at 37°C for 3 h, followed by purification under denaturing conditions, as previously described [30]. Afterward, rTsCstN was dialyzed against 1 × PBS to remove urea and imidazole as well as refold protein. Expression and purification of rTsCstN were determined by 15% SDS-PAGE and confirmed by western blot analysis reacting with mouse anti-His tag antibody (Ab) (BioLegend, San Diego, CA). The contaminated endotoxin was removed using Pierce High-Capacity Endotoxin Removal Resin (Thermo Fisher Scientific), and the residual endotoxin was assessed by Pierce LAL Chromogenic Endotoxin Quantitation Kit (Thermo Fisher Scientific), which needed to be less than 0.5 EU/ml according to the endotoxin normative standard as described by the United States Food and Drug Administration [31]. The endotoxin concentration of rTsCstN was determined to be 0.078 EU/ml. rTsCstN was used for functional analysis and immunization of mice to produce hyperimmune serum as mentioned previously with some modifications [32]. In brief, five female ICR mice, six to eight-week-old, were intraperitoneally immununized with 20 μg of rTsCstN emulsified with Imject Alum Adjuvant (Thermo Fisher Scientific), followed by two boosts with the same amount of protein emulsified with Alum at 14-day interval. Seven days after the final boost, blood were collected by heart puncture and sera were stored at -20°C for further experiments.

### Western blot analysis

ES products of L1 prepared as mentioned above and crude worm antigens (CWA) of L1 prepared as previously described [33] were loaded into 12% SDS-PAGE and transferred onto a PVDF membrane (Pall Corporation, Port Washington, NY). The membrane was incubated in a blocking solution [5% (w/v) skim milk in PBST (1 × PBS containing 0.05% Tween 20)] at RT for 1 h and subsequently incubated with a 1:100 mouse anti-rTsCstN serum or 1:100 pre-immunized serum at 4°C, overnight. After washing thoroughly with PBST, the membrane was incubated with 1:3,000 horseradish peroxidase (HRP)-conjugated goat anti-mouse IgG (Southern Biotech, Birmingham, LA) at RT for 1 h. For detection, the membrane was incubated with a 2,6-dichloroindophenol substrate (Sigma-Aldrich Co., St. Louis, MO) until the reacted protein band appeared. The reaction was stopped by washing several times with ddH_2_O.

### Immunolocalization of TsCstN in parasites

*T*. *spiralis*-infected mice muscles were obtained and immediately fixed in fixing solution (4% paraformaldehyde, 1 × PBS, pH 7.4) before the preparation of paraffin-embedded sections and immunolocalization as previously described with some modifications [34]. In summary, 3-μM-thick sections were deparaffinized, rehydrated, retrieved antigenic epitopes and inactivated endogenous peroxidase before blocking with 3% FBS in 1 × PBS at RT for 1 h. Subsequently, the sections were incubated overnight with the 1:100 mouse anti-TsCstN serum or 1:100 pre-immunized serum at 4°C. After washing thrice with PBST, the sections were overlaid with 1:1,000 HRP-conjugated goat anti-mouse Ig (Southern Biotech) and incubated at RT, for 1 h. The chromogenic result was developed using AEC staining kit (Sigma-Aldrich) according to the manufacturer’s instructions. The slides were examined using a light microscope.

### Protease inhibitor assay

The protease inhibition capacity of rTsCstN was performed against human cathepsin (Cat) B (Sigma-Aldrich), human CatL (Sigma-Aldrich), human CatS (Sigma-Aldrich), and trypsin (Sigma-Aldrich). The assay was prepared as described previously with some modifications [17]. In summary, rTsCstN was preincubated in an assay buffer (100 mM sodium acetate, pH 5.5, 100 mM NaCl, 1 mM DTT) at 37°C for 30 min before adding CatB (0.5 μM), CatL (0.05 nM), CatS (0.005 nM), or trypsin (0.5 μM). The fluorogenic substrate of CatB (20 mM of benzyloxycarbonyl (Z)-Arg-Arg-aminoethylcoumarine (AMC), Sigma-Aldrich) and CatL and CatS (20 mM of Z-Phe-Arg-AMC, Sigma-Aldrich), or the colorimetric substrate of trypsin (1 mM Nα-benzoyl-L-arginine 4-nitroanilide hydrochloride; Sigma-Aldrich) was added into each reaction mixture, and the mixture was additionally incubated at 37°C. The fluorescence intensity was monitored using fluorometer (Synergy H1 Hybrid Reader, BioTek, BioTek, Winooski, VT) with excitation at 360 and emission at 460 nm, and an optical density was monitored at a wavelength of 405 using a Sunrise microplate reader (Tecan Group Ltd., Männedorf, Switzerland). The concentration of rTsCstN at 50% inhibition of enzyme activity (IC50) was determined by nonlinear regression analysis.

To determine the inhibition of rTsCstN over CatL in mBMDMs lysate, LPS-treated mBMDMs were resuspended in lysis buffer (50 mM NaH_2_PO_4_, 300 mM NaCl, pH 8.0) before sonication and centrifugation at 16,000 × g for 30 min, at 4°C. The supernatant was obtained, and then the concentration of CatL was measured by hydrolysis of its fluorogenic substrate while comparing with a standard CatL concentration. Of these, mBMDMs lysate containing approximately 0.17 nM CatL was then incubated with the varying concentrations (0.17 nM to 5 μM) of rTsCstN at 37°C for 30 min in an assay buffer. The enzyme activity was measured after adding the fluorogenic substrates (20 mM of Z-Phe-Arg-AMC, Sigma-Aldrich). E64 (Sigma-Aldrich) and rmDHFR were used as positive and irrelevant controls, respectively.

### Internalization of rTsCstN into mBMDMs

One ml of progenitor cells isolated from mouse bone marrow were plated at an intensity of 5 × 10^5^ cells/ml onto 10% poly-L lysine-coated coverslips in 12-well culture plates (Corning Inc., Corning, NY). After differentiation for 7 days, the mBMDMs were treated with LPS under 5% CO_2_ for 12 h at 37°C, followed by incubation with 2.5 μg/ml FITC-labelled rTsCstN. rTsCstN was labeled with FITC using EZLabel Protein FITC Labeling Kit (BioVision, Milpitas, CA) according to the manufacturer’s protocol. Treatment with FITC-labelled rmDHFR or culture media was used as a negative control. After incubation for 1 h, the cells were washed three times with ice-cold PBS, fixed with 4% paraformaldehyde, and permeabilized by incubating with 0.02% Tween 20 in PBS for 10 min. After blocking with 5% FBS in PBS, the lysosome was tracked with 1:1,000 rabbit anti-human LAMP1 IgG (Millipore, Temecula, CA) for 1 h and subsequently incubated with 1:2,000 Cy3-conjugated donkey anti-rabbit IgG (Biolegend) for 1 h. After washing three times with PBS, the nuclei were counterstained with Hoechst 33342 (Invitrogen) for 20 min. The coverslips were washed several times with PBS and then mounted with FluorSave reagent (Calbiochem, Darmstadt, Germany). The localization was determined by observing the staining patterns with a ×100 oil objective lens on a laser scanning confocal microscope (LSM700, Zeiss, Jena, Germany). Digital images were captured using the Zeiss microscope software package ZEN 2012 (Zeiss).

### Statistical analysis

Statistical analyzes were conducted using GraphPad Prism 6 software (GraphPad Software Inc., La Jolla, CA). The significance of the differences between groups was analyzed using the one-way ANOVA. Individual data and mean ± SD of the group are presented. A *p*-value < 0.05 was considered statistically significant.

## Results

### Effects of ES fractions on LPS-treated mBMDMs

**Fig 1:**
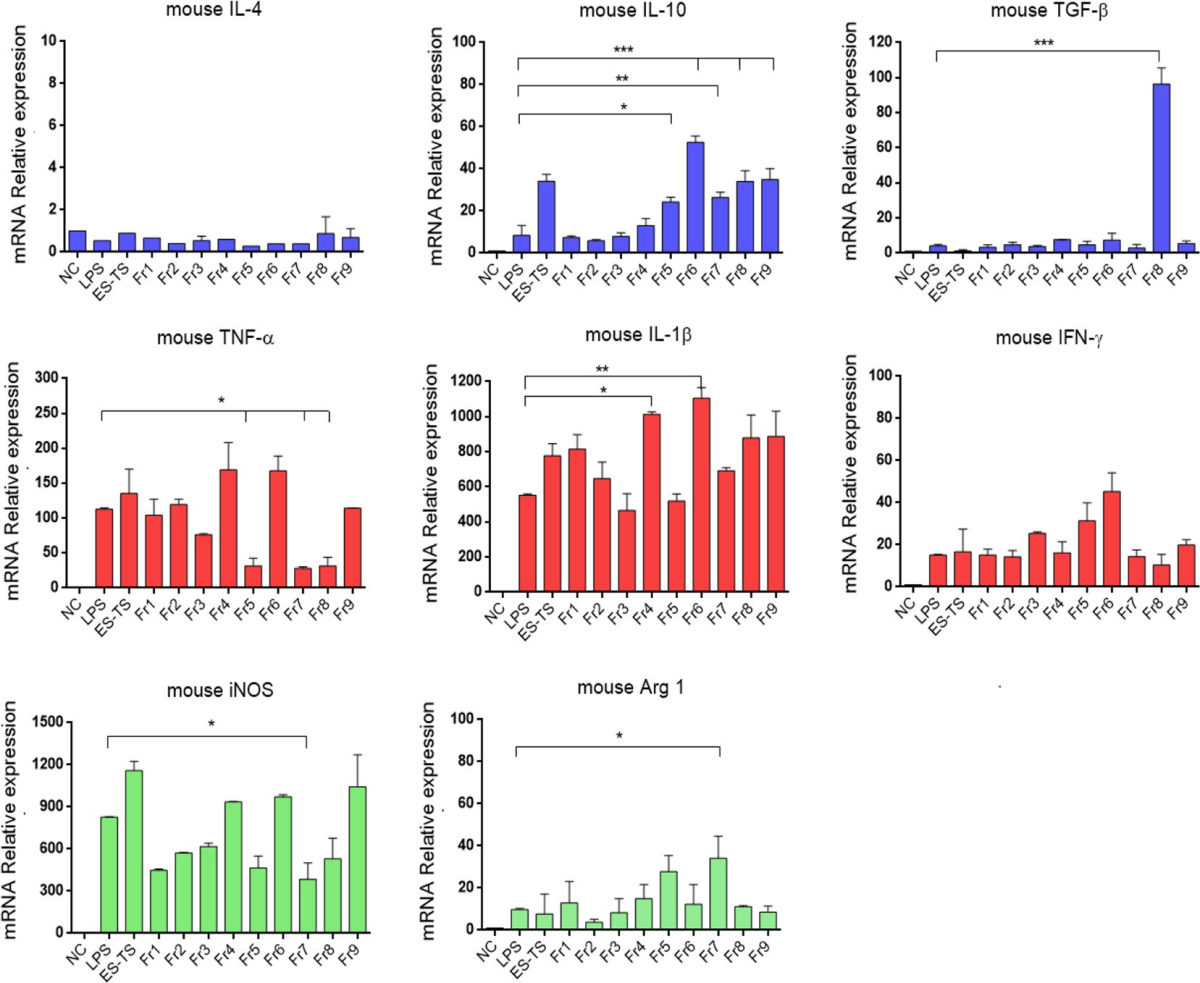
Transcription of cytokines and M1/M2 markers after treatment of mBMDMs with ES-L1 fractions. mBMDMs were treated with each ES-L1 fraction (10 μg/ml) for 24 h before LPS stimulation (100 ng/ml) for 12 h. The cells were harvested, and the transcription levels of mRNA cytokines and M1/M2 markers were determined using qRT-PCR. Negative control (NC) was the medium alone. Glyceraldehyde-3-phosphate dehydrogenase (GAPDH) was used as a housekeeping gene for normalization. Each color of the bar graph is explained: “Blue” Anti-inflammatory cytokines, “Red” Pro-inflammatory cytokines, and “Green” M1/M2 markers. The results are expressed as mean ± SD. The experiments were performed in triplicate with two independent experiments. One-way ANOVA followed by a Bonferroni multiple comparison test were used for analysis: *p < 0.05, **p < 0.01, and ***p < 0.001, represent differences between LPS-stimulated mBMDM treated with each ES-L1 fraction and LPS-stimulated-only mBMDMs.

Fractionation of ES-L1 using anion exchange chromatography demonstrated that there were nine fractions containing protein bands with different molecular weights (S1 Fig). Each protein fraction was incubated with mBMDM before LPS stimulation, and thereafter, expression of cytokines was monitored using qRT-PCR. The results showed that transcription of anti-inflammatory IL-10 was significantly induced by ES-L1 and fractions 5–9; with fraction 6 inducing the highest levels of IL-10 expression. However, TGF-β was not stimulated by ES-L1 but significantly upregulated when treated with fraction 8. The expression of IL-4 was neither detected in LPS alone or combinations with ES-L1 or ES-L1 fractions. IFN-γ was increased after induction with LPS but could not be suppressed when treated with ES-L1 or the fractions (Fig 1).

Fractions 5, 7, and 8 markedly downregulated the expression of TNF-α, and fractions 3 and 5 downregulated the expression of IL-1β. The macrophage phenotypic markers, iNOS and Arg-1, which correlate to the M1 and M2 macrophage phenotypes, respectively, were also determined, and it was apparent that fraction 7 significantly suppressed the expression of iNOS and upregulated the expression of Arg-1 as compared with LPS-only treatment. Based on the above profiles, we decided to select fractions 5–8 (molecular weight range 7–50 kDa) for further analysis by mass spectrometry.

After mass spectrometry analysis, six different proteins were identified in fractions 5–8; the results are presented in Table 1. Conserved hypothetical protein Tsp_04814 (accession no. gi|339258426) presented the highest MS score and the number of matched peptides. Therefore, conserved hypothetical protein (Ts_04814) was considerably chosen as the first target to evaluate its role in mBMDM immunomodulation.

**Table 1:** Identification of proteins in ES-L1 fractions 5–8 using Nano-LC/MS/MS. The experiments were performed in triplicate.

Fraction no.	Protein name	Accession no.	Theoretical ^b^		MS ^c^ score	No. matched peptide ^d^
			Mw	pI		
5	Conserved hypothetical protein ^a^ (Tsp_00135)	gi|339239909	119.2	6.18	15	1
6	Conserved hypothetical protein ^a^ (Tsp_04814)	gi|339258426	13.2	4.54	328	4
7	Conserved hypothetical protein ^a^ (Tsp_04814)	gi|339258426	13.2	4.54	158	3
	ATP-dependent RNA helicase DDX19B	gi|339241083	14.9	4.6	265	1
8	ATP-dependent RNA helicase DDX19B	gi|339241083	14.8	4.6	198	2
	Hypothetical protein ^a^ (Tsp_04680)	gi|339258142	36.3	4.75	181	2
	Conserved hypothetical protein ^a^ (Tsp_16075)	gi|339255022	13.9	4.75	48	1
	Antigen targeted by protective antibodies	gi|404638	31.2	4.76	135	2

^a^ Locus tag of protein

^b^ Theoretical molecular mass (kDa) and isoelectric point (pI).

^c^ A MS score obtained from MASCOT search results of >70 was used to assign identity to a protein.

^d^ Number of matched peptide was the significant matched protein sequence.

### Bioinformatics analysis exhibits a function of the conserved hypothetical protein (Ts_04814) as cystatin

**Fig 2:**
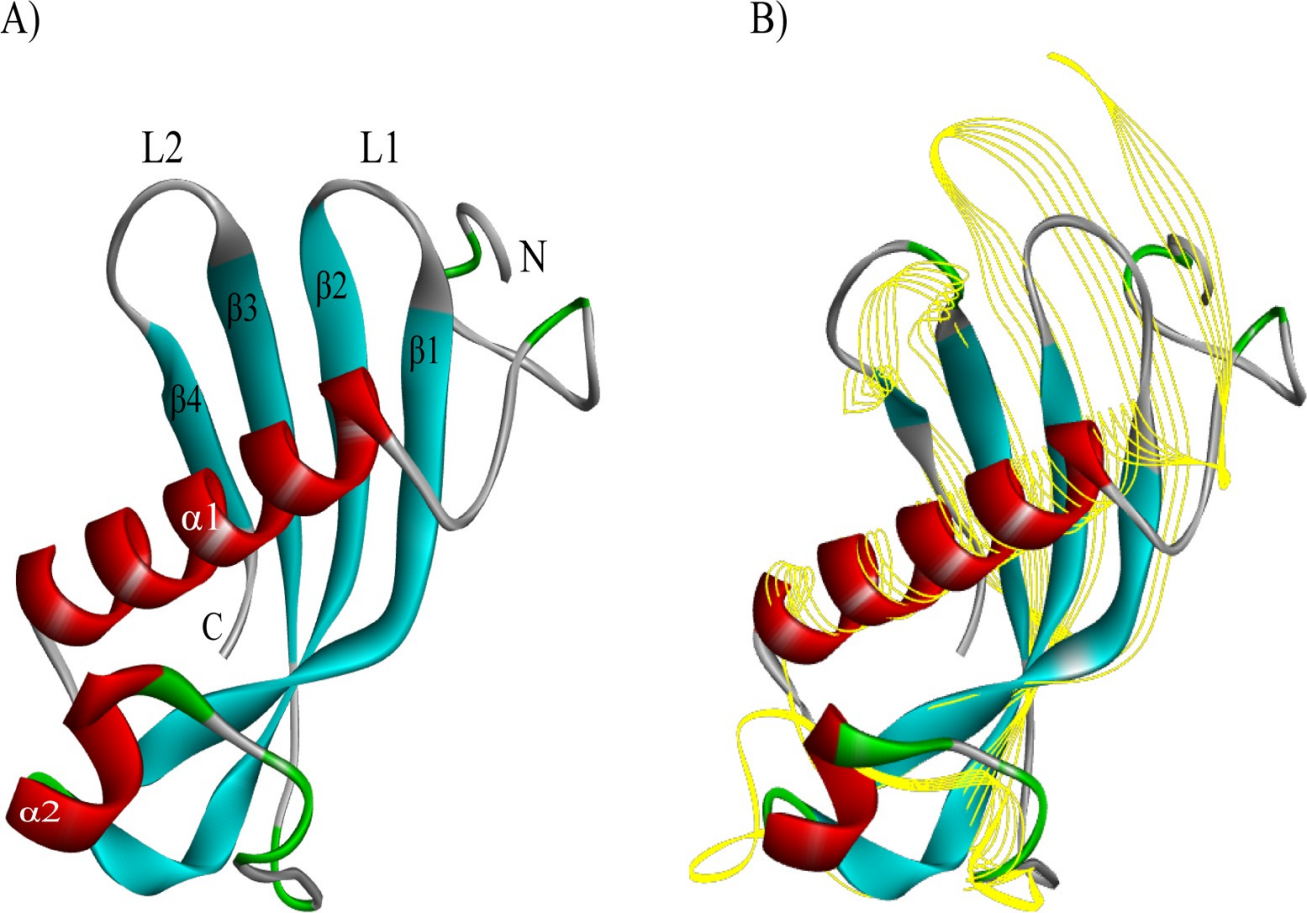
3D structural comparison of conserved hypothetical protein (Ts_04814) with human cystatin E/M using the I-TASSER program. (A) The predicted tertiary structure of the conserved hypothetical protein (Ts_04814); there are 2 α-helixes (in red), 4 β-strands (in blue), and irregular coils (in green). (B) The structural comparison between conserved hypothetical protein (Ts_04814) (solid ribbon) and human cystatin E/M (PDB ID. 4n6o) (yellow line ribbon).

The 3D structure of TsCstN was simulated using several templates selected by I-Tasser. The top 10 threading templates were provided in Supplementary S2 Table. The 3D structure of the conserved hypothetical protein (Ts_04814) is composed of an N-terminal region, four β-sheets forming two hairpin loops (L1 and L2), the loop 1 (between β1 and β2), and the loop 2 (between β3 and β4) and two α-helix, with the α1 located at the center of the structure (Fig 2A). After structural alignment, the results demonstrated that conserved hypothetical protein (Ts_04814) has the closest structural similarity to human cystatin E/M (PDB ID. 4n6o) (Fig 2B), which presented the highest TM score (TM score = 0.690; S3 Table). Therefore, conserved hypothetical protein (Ts_04814) was postulated to function as cystatin E/M and was subsequently designated as a *T*. *spiralis* novel cystatin (TsCstN).

**Fig 3:**
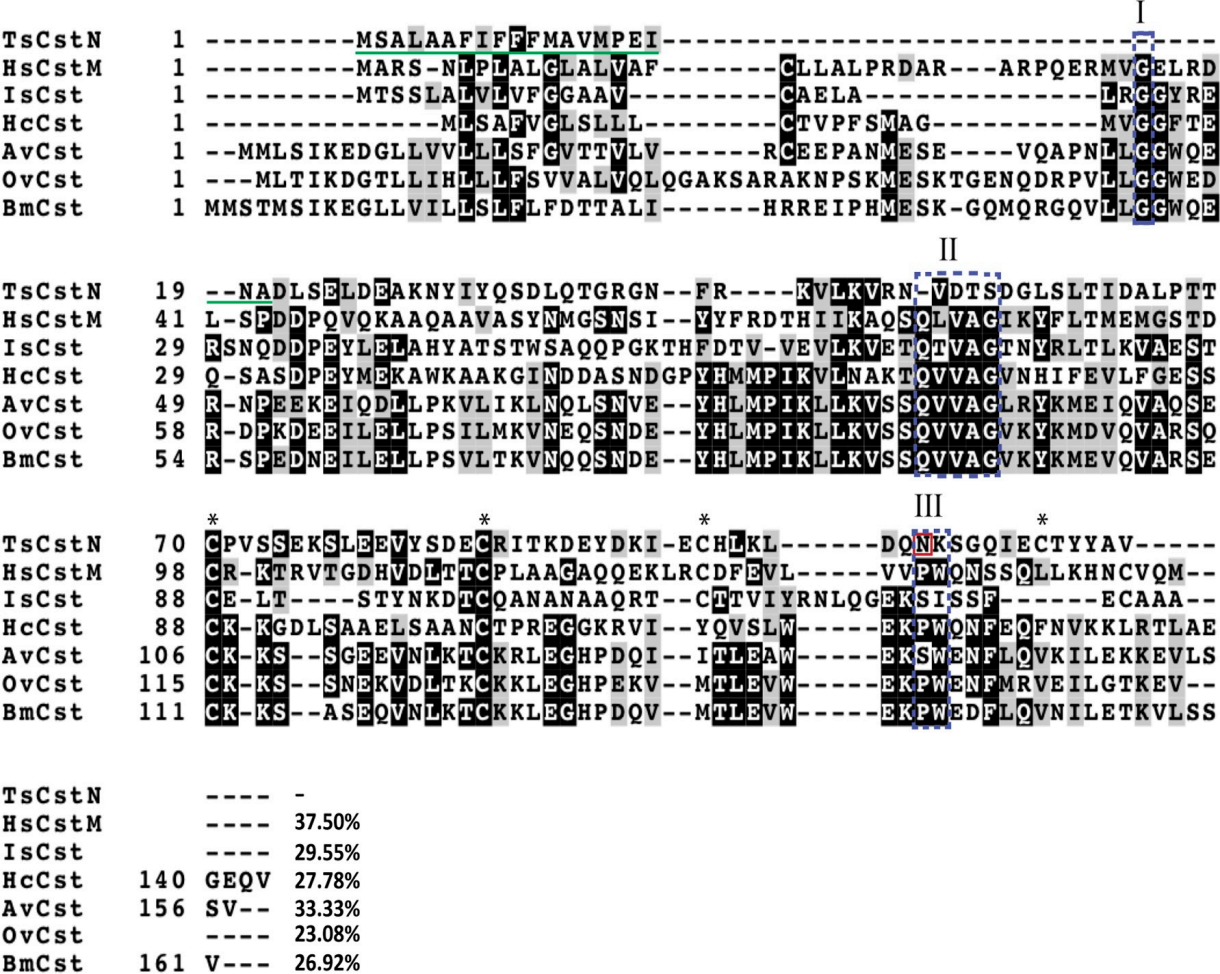
Multiple alignments of TsCstN with other type 2 cystatin homologs. Amino acid sequences were aligned and displayed using Clustal Omega and BoxShade server (https://embnet.vital-it.ch/software/BOX_form.html), respectively. HsCstM of human (accession no. NP_001314), IsCst of *Ixodes scapularis* (accession no. XP_002434439), HcCst of *Haemonchus contortus* (accession no. CDJ92568), AvCst of *Acanthocheilonema viteae* (accession no. L43053), OvCst of *Onchocerca volvulus* (accession no. P22085), and BmCst of *Brugia malayi* (accession no. AAB69857). Conserved cystatin motifs are composed of N-terminal conserved glycine motif (Box I), QXVXG motif (Box II), and PW motif (Box III). The signal peptide of TsCstN is in green underline. Four cysteine residues are indicated by asterisks (*), and the potential N-glycosylation site of TsCstN is indicated by a red box. %identity between TsCstN and its homologs are indicated at the end of sequence.

To predict the basic properties of TsCstN protein, different bioinformatics programs as mentioned in the Materials and Methods were used for analysis. The complete coding sequence of TsCstN, obtained *via* the NCBI database, extends 354 nucleotides and encodes for a protein of 117 amino acid (aa) residues with a theoretical molecular mass of 13.2 kDa and an isoelectric point (pI) of 4.54. The TsCstN protein contains a signal peptide between aa residues 1–20 (MSALAAFIFFFMAVMPEINA), but did not present a transmembrane helix, which indicates that TsCstN is a secretory protein that is facilitated through the classical secretory pathway. Predictions of glycosylation site and disulfide bond suggested that TsCstN contains one potential N-glycosylation site at N_105_ and presents four cysteine residues at C_70_, C_86_, C_98_, and C_112_, which potentially form two disulfide bonds, including C_70_–C_112_ and C_86_–C_98,_ respectively (Fig 3).

Conserved motif and consensus residues of the cystatin family were identified in TsCstN by comparing the multiple sequence alignment with that of its homologs (Fig 3). The alignment exhibited that TsCstN does not contain conserved sequences of the type 2 cystatins, including N-terminal conserved glycine (region I), QXVXG conserved motif (region II), and PW conservative site (region III) (Fig 3). However, the detailed functions of TsCstN would be evaluated using recombinant protein technology and inhibition of papain-like cysteine proteases as described below.

### Expression of rTsCstN and detection of native TsCstN

**Fig 4:**
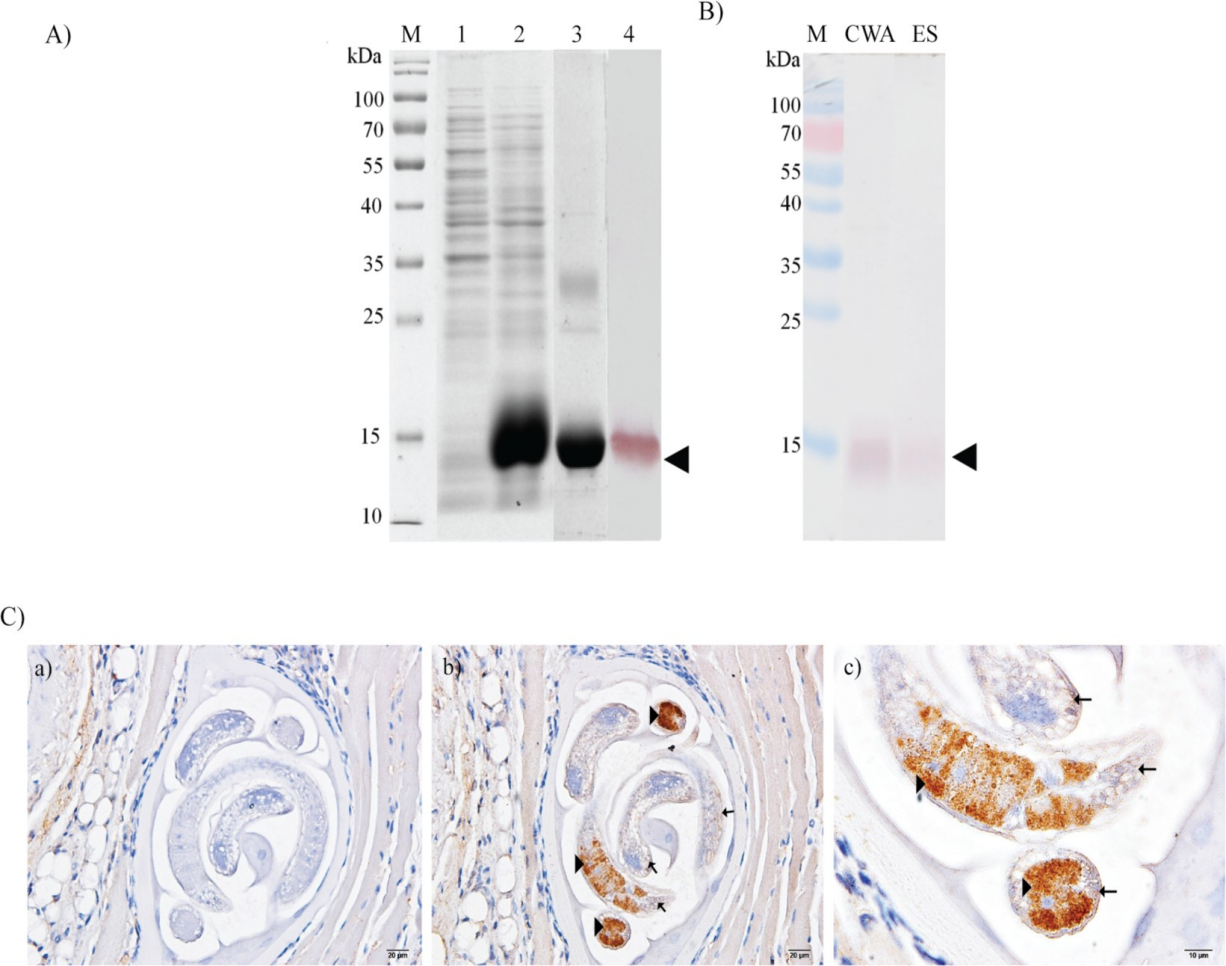
Expression of recombinant TsCstN and detection of native TsCstN in parasite antigens and tissue. (A) rTsCstN was successfully expressed in *Escherichia coli*. M, PageRuler Prestained Protein Ladder (Thermo Scientific); 1, soluble protein fraction; 2, insoluble protein fraction; 3, purified rTsCstN; and 4, western blot analysis of rTsCstN probed with anti-His tag antibody. Arrowhead indicates rTsCstN. (B) Western blot analysis detecting native TsCstN in CWA and ES products of *T*. *spiralis* L1 using mouse anti-rTsCstN serum. M, PageRuler Prestained Protein Ladder (Thermo Scientific); CWA, crude worm antigen of L1; ES, excretory–secretory products of L1. Arrowhead indicates native TsCstN. (C) Immunolocalization of TsCstN in the tissue of *T*. *spiralis* L1. Paraffin-embedded sections of *T*. *spiralis*-infected mice muscles were used for determination of TsCstN expressed in the parasite tissue. Control tissue incubated with mouse pre-immune serum (a), *T*. *spiralis* L1 tissue incubated with mouse anti-rTsCstN serum (100×) (b) and higher magnification (400×) (c). Arrowheads indicate localization of TsCstN at the stichosome, and arrows indicate localization of TsCstN at subcuticular cells.

A full-length TsCstN cDNA lacking a signal peptide was amplified and subsequently subcloned into a prokaryotic expression system for recombinant protein expression. rTsCstN was successfully overexpressed in an insoluble form at a molecular weight of approximately 13 kDa. After purification and refolding, purified rTsCstN could solubilize and reacted with mouse anti-His tag Ab (Fig 4A). The conformation of rTsCstN was analyzed using Circular Dichroism (CD) spectrometry, revealing that rTsCstN formed a secondary structure identical to that of other type 2 cystatins [35] (S2 Fig). Polyclonal mouse anti-rTsCstN serum level was increased and could detect native TsCstN in CWA and ES of L1 at an MW of approximately 13 kDa similar to that of rTsCstN (Fig 4B). Immunolocalization of native TsCstN in L1 tissue using mouse anti-rTsCstN serum demonstrated that TsCstN was predominantly localized at stichosome and subcuticular cells. Control tissues probed with pre-immune sera revealed no reactivity (Fig 4C).

### rTsCstN is functionally active

**Fig 5:**
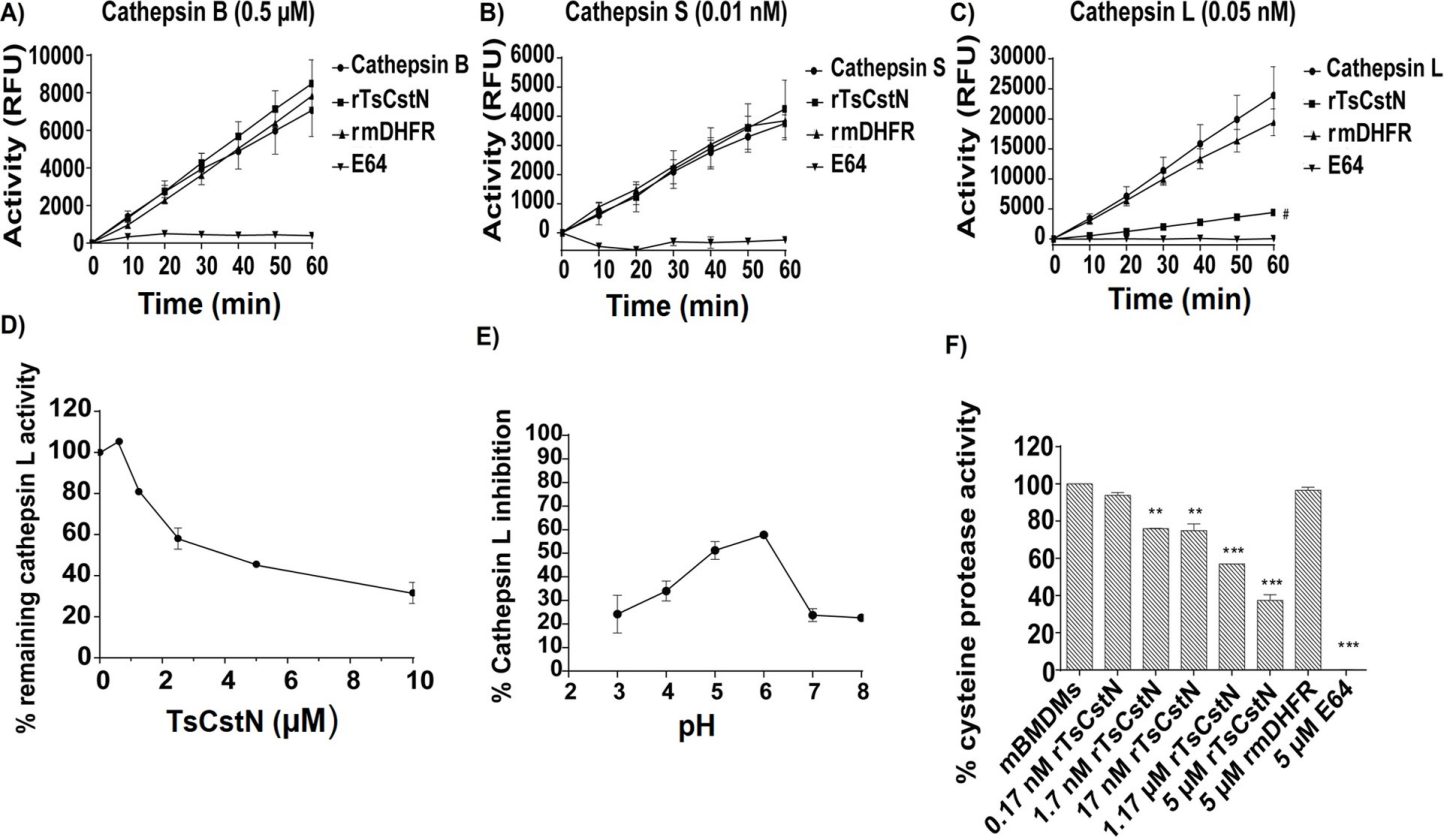
Inhibitory activity of rTsCstN against Cat B (A), Cat S (B), and Cat L (C) demonstrated that rTsCstN specifically inhibited CatL activity but not that of CatB and CatS. rTsCstN could inhibit CatL activity in a dose-dependent manner (D). rTsCstN could inhibit CatL activity at the maximum at pH 6, followed by pH 5 (E). rTsCstN partially inhibited cysteine protease activity in mBMDM lysate to hydrolyze a fluorogenic substrate of CatL and CatS, Z-Phe-Arg-AMC, in a dose-dependent manner (F). E64 inhibitor, used as a positive control, completely inhibited CatB, CatS, and CatL activities. Irrelevant control (rmDHFR) could not inhibit any of CatB, CatS, and CatL activities. The result reported as mean ± SD. The experiments were performed in triplicate with three independent experiments. One-way ANOVA followed by a Bonferroni multiple comparison test were used for analysis: #*p* < 0.05, represents difference between CatL activity inhibited by rTsCstN and CatL activity only; **p < 0.01, and ***p < 0.001, represent differences between mBMDM lysate treated with dose-dependent manner of rTsCstN and cysteine protease activity in mBMDM lysate only.

To evaluate the inhibitory properties of rTsCstN over papain-like cysteine proteases, rTsCstN was incubated with the papain-like cysteine proteases, including cathepsin (Cat) B, CatL, and CatS, before hydrolysis of fluorogenic substrate. The results demonstrated that rTsCstN effectively inhibited CatL, but not CatB and CatS (Fig 5A–5C). A recombinant mouse dihydrofolate reductase (rmDHFR) used as an irrelevant control protein could not inhibit the activity of any cathepsin. E64, the broad-spectrum cysteine protease inhibitor, completely inhibited CatB, CatL, and CatS. Cross-class inhibition testing of rTsCstN on the serine protease, trypsin, was conducted and revealed that rTsCstN did not inhibit trypsin activity (S3 Fig).

The IC_50_ value of rTsCstN required to inhibit 50% activity of CatL (Fig 5D) was calculated to be 3.88 μM. pH-dependent assays suggested that rTsCstN (concentration at 10 μg/ml) could inhibit maximum hydrolysis activity of CatL at pH 6 (58%) followed by pH 5 (50%). The inhibitory activity of rTsCstN was decreased when incubated at pH 3, 7, and 8 (Fig 5E). The optimal pH that facilitates the maximum inhibitory activity of rTsCstN ranged from pH 5–6. Moreover, we examined whether rTsCstN directly inhibited cysteine proteases in mBMDMs. The results confirmed that rTsCstN could partially inhibit cysteine protease hydrolyzing Z-Phe-Arg-AMC, a fluorogenic substrate of CatL and CatS, in a dose-dependent manner, whereas rmDHFR and E64 showed negative and complete inhibition of substrate hydrolysis, respectively (Fig 5F).

### mBMDMs internalizes rTsCstN

**Fig 6:**
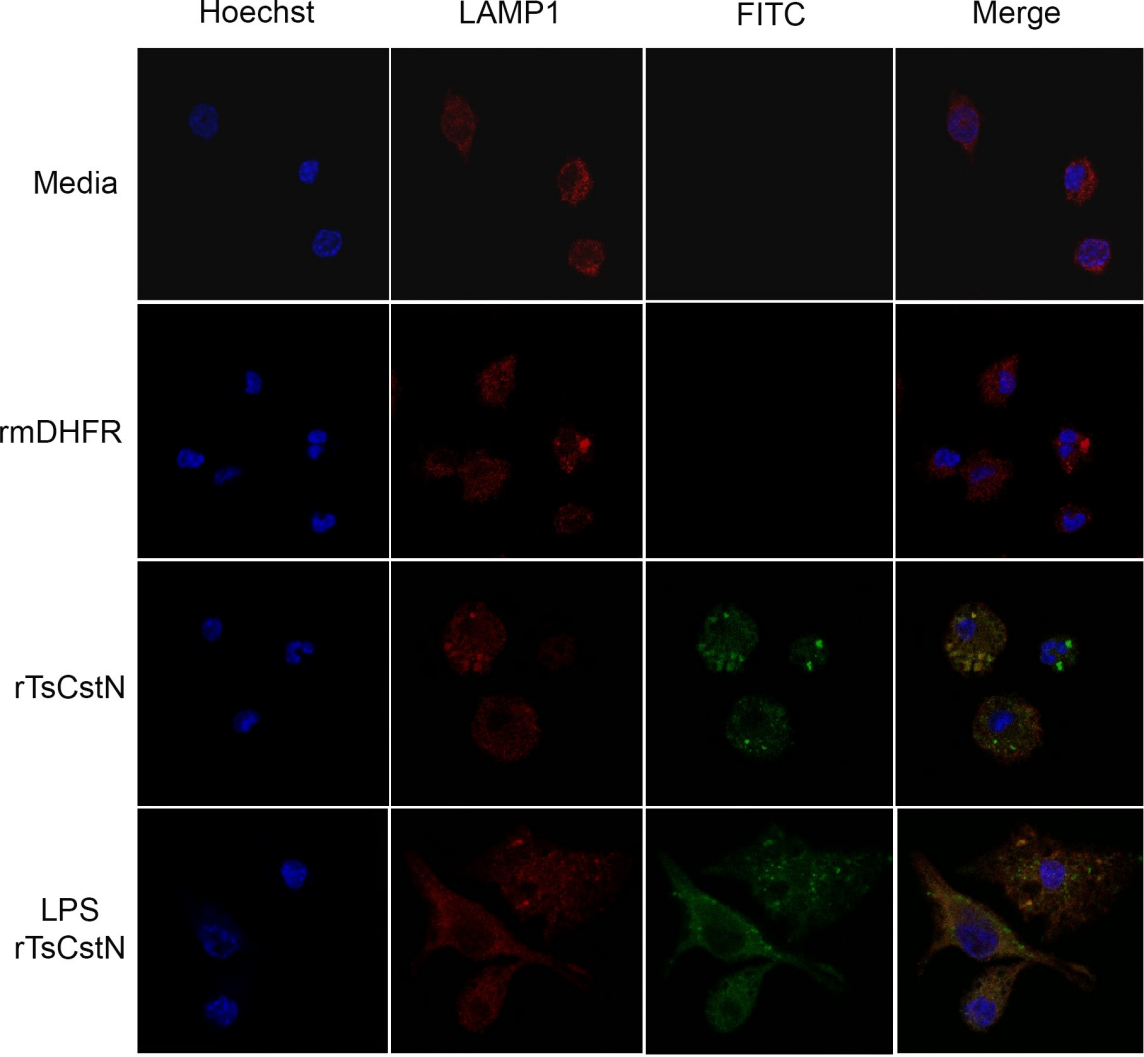
mBMDMs internalizing rTsCstN was determined using a laser scanning confocal microscope. rTsCstN (green) were mainly detected in the cytoplasm of both untreated and LPS-treated mBMDMs. Moreover, rTsCstN were co-localized with CatL-containing lysosomes (yellow). Lysosomes were labeled with rabbit anti-human LAMP1 IgG plus Cy3-conjugated donkey anti-rabbit IgG (red), and the nucleus was counterstained with Hoechst 33342 (blue). Culture media- or FITC-labelled irrelevant control protein (rmDHFR) were used as negative controls.

The internalization of rTsCstN into mBMDMs was investigated using immunofluorescence confocal microscopy. FITC-tagged rTsCstN was incubated with mBMDM, and rTsCstN was found within cells after 60 min of incubation, mostly accumulating in the cytoplasm. Some protein was found to be co-localized with the lysosome (stained red by LAMP1), in both LPS-stimulated mBMDMs and non-treated controls (yellow) (Fig 6). Z-stack analysis confirmed internalization of rTsCstN into mBMDM and co-localization between rTsCstN and lysosome (S4 Fig).

### rTsCstN inhibited pro-inflammatory cytokines produced from LPS-stimulated mBMDMs

**Fig 7:**
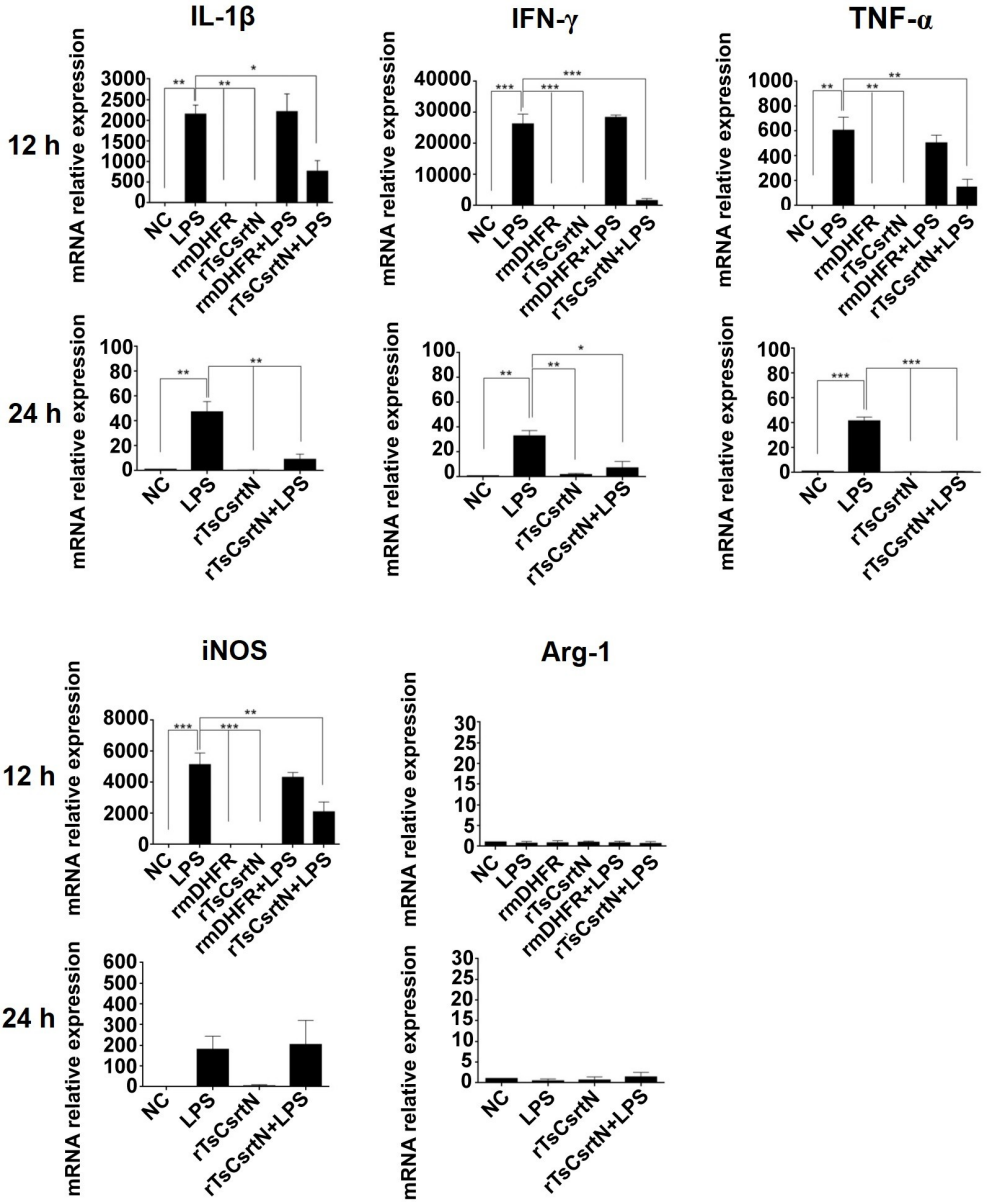
rTsCstN suppressed inflammation mediated by LPS-stimulated mBMDMs. Transcription levels of IL-1β, IFN-γ, TNF-α, and iNOS were downregulated when LPS-stimulated mBMDMs were treated with rTsCstN after incubation for 12 and 24 h. Irrelevant control (rmDHFR) could not downregulate IL-1β, IFN-γ, TNF-α, and iNOS levels mediated by LPS-stimulated mBMDMs. rTsCstN did not affect the level of anti-inflammatory cytokines and M2 marker (S5 Fig). Negative control (NC) was the medium alone. The results are expressed as mean ± SD. The experiments were performed in triplicate with three independent experiments. One-way ANOVA followed by a Bonferroni multiple comparison test were used for analysis: *p < 0.05, **p < 0.01, and ***p < 0.001, represent differences of cytokine mRNA between LPS-stimulated mBMDM treated with rTsCstN and LPS-stimulated-only mBMDMs.

**Fig 8:**
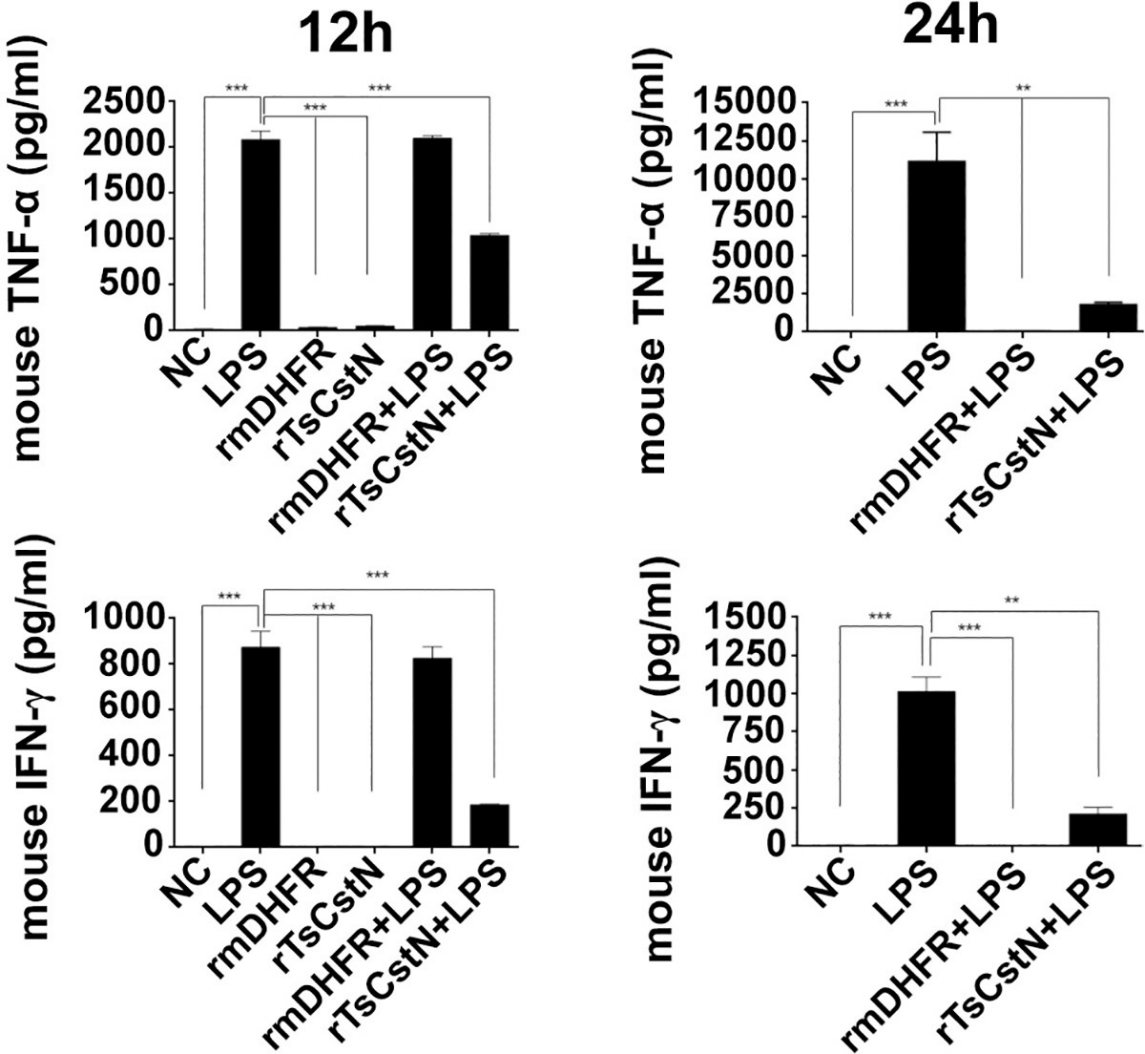
Production of the pro-inflammatory cytokines, TNF-α and IFN-γ, was determined in a culture medium of LPS-treated mBMDMs in the presence or absence of rTsCstN. After measuring the cytokine levels by sandwich ELISA, TNF-α and IFN-γ levels were significantly decreased when we treated LPS-stimulated mBMDMs with rTsCstN after incubation for 12 and 24 h. Irrelevant control (rmDHFR) showed no inhibition of the level of TNF-α and IFN-γ produced by LPS-stimulated mBMDMs. Negative control (NC) was the medium alone. The results are expressed as mean ± SD. The experiments were performed in triplicate with three independent experiments. One-way ANOVA followed by a Bonferroni multiple comparison test were used for analysis: *p < 0.05, **p < 0.01, ***p < 0.001, represent differences of cytokine production between LPS-stimulated mBMDM treated with rTsCstN and LPS-stimulated-only mBMDMs.

To determine immunomodulatory property of TsCstN, LPS-stimulated BMDMs were treated with rTsCstN followed by monitoring cytokine levels using qRT-PCR and ELISA. For the mRNA expression, treatment with rTsCstN significantly downregulated pro-inflammatory cytokines, IL-1β, IFN-γ, and TNF-α of LPS-stimulated mBMDMs after 12 h incubation, and this reduction was maintained until 24 h. rmDHFR could not suppress the expression of pro-inflammatory cytokines in LPS-stimulated mBMDMs (Fig 7). Production of pro-inflammatory cytokines, IFN-γ and TNF-α, was determined in culture medium using ELISA to confirm mRNA expression. The results demonstrated that LPS-stimulated mBMDMs treated with rTsCstN significantly decreased the production of IFN-γ (*P* < 0.001) and TNF-α (*P* < 0.01) at 12 and 24 h, respectively (Fig 8). In contrast, anti-inflammatory cytokine of both mRNAs (IL-4, IL-10, and TGF-β) and proteins (IL-4 and IL-10) exhibited no significant difference between rTsCstN-treated + LPS-stimulated mBMDMs and LPS-stimulated-only mBMDMs (*P* > 0.05) (S5 Fig).

To investigate the macrophage phenotypes, mRNA levels of iNOS (M1 phenotype) and Arg-1 (M2 phenotype) were determined in rTsCstN-treated + LPS-stimulated mBMDMs. Expression of iNOS was significantly downregulated in rTsCstN-treated + LPS-stimulated mBMDMs when compared with untreated control at 12 h (*P* < 0.01), whereas mRNA expression of Arg-1 was not altered under any treatment conditions (Fig 7).

### rTsCstN inhibited expression of MHC class II but not costimulatory molecules (CD80 and CD86) in LPS-stimulated mBMDMs

**Fig 9:**
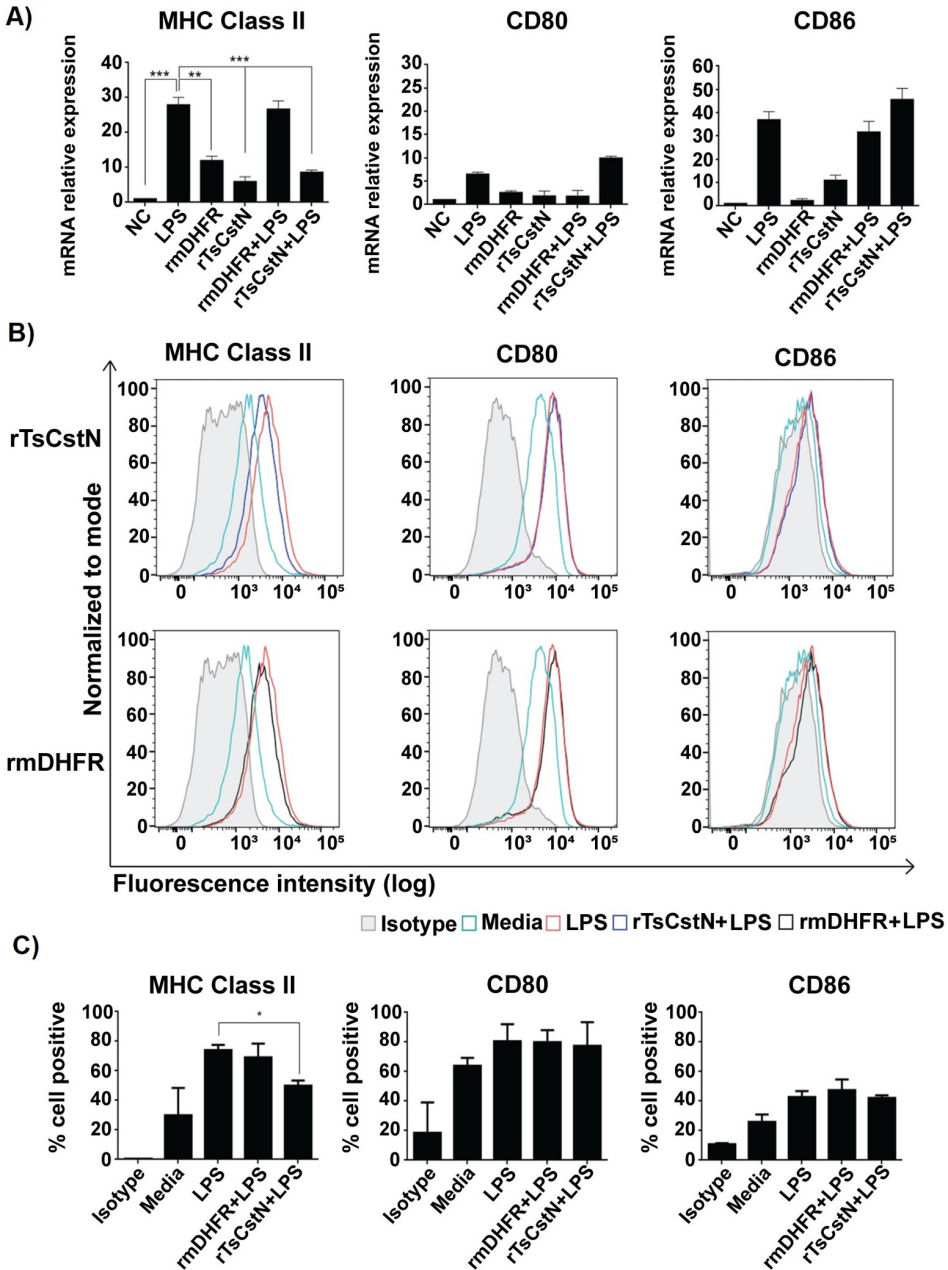
mBMDMs were cultured with or without rTsCstN for 24 h and subsequently stimulated with LPS for 12 h. Treatment with rTsCstN suppressed mRNA expression of MHC-II, but not its costimulatory molecules (CD80 and CD86) (A). Surface expression of MHC-II, CD80, and CD86 of and costimulatory molecules (CD80 and CD86) on LPS-stimulated mBMDMs was determined by flow cytometry. FACS histograms from one representative experiment suggested that rTsCstN could interfere with surface expression of MHC-II, but not that of CD80 and CD86 (B). The percentage of MHC-II, CD80, or CD86 positive cells on the mBMDMs was calculated, and this emphasized the impact of rTsCstN on suppression of MHC-II expression (C). The data were determined by Flowjo software (TreeStar, Ashland, OR). Two of the three experiments were presented in the form of the percentage of positive cells. Treatment with irrelevant control (rmDHFR) showed a negative result. The results are expressed as mean ± SD. The experiments were performed in triplicate with three independent experiments. One-way ANOVA followed by a Bonferroni multiple comparison test were used for analysis: *p < 0.05, **p < 0.01, and ***p < 0.001, represent differences between LPS-stimulated mBMDM treated with rTsCstN and LPS-stimulated-only mBMDMs.

In previous studies, recombinant onchocystatin (rOv17) derived from *O*. *volvulus* reduced the expression of MHC class II (MHC-II) and the costimulatory molecule CD86 on human monocytes, which suppressed host immune response against the parasite [36]. We showed here that treatment with rTsCstN significantly suppressed the mRNA expression of MHC-II in LPS-stimulated mBMDMs compared with LPS-only treatment (*P* < 0.001). Treatment of LPS-stimulated mBMDMs with rmDHFR did not reduce mRNA expression of MHC-II (*P* > 0.05) (Fig 9A). For costimulatory molecules, mRNA expressions of CD80 and CD86 showed no significant change in rTsCstN-treated + LPS-stimulated mBMDMs when compared with LPS-only treatment (*P* > 0.05) (Fig 9A). However, mRNA expression of CD80 and CD86 significantly increased even after rTsCstN-only or rmDHFR-only treatments compared with that of the negative control group (media only).

Surface marker expression of MHC-II, CD80, and CD86 on mBMDM was also evaluated using FACS and the result confirmed that expression of MHC-II, but not CD80 and CD86, was significantly suppressed in LPS-stimulated mBMDMs treated with rTsCstN compared with that of LPS-only treatment (*P* ≤ 0.05) (Fig 9B and 9C).

## Discussion

Cystatins that function as papain-like cysteine protease inhibitors are classified into three major families comprising type 1 cystatins or stefins, characterized by 11 kDa unglycosylated intracellular inhibitors without signal peptide and disulfide bond; type 2 cystatins, with molecular masses of 13 to 14 kDa and possessing signal peptide and disulfide bridges, which, in some members, are glycosylated; and type 3 cystatins or kininogens, characterized by molecular masses ranging from 88 to 114 kDa and glycosylation of three family 2 cystatin domains [37]. Besides protease inhibition, cystatins have been characterized as immunomodulatory molecules, especially in parasitic helminths. Filarial cystatins, including CPI-2, isolated from *Brugia malayi*; onchocystatin, from *Onchocerca volvulus*; and AvCystatin, from *Acanthocheilonema viteae*, could regulate immune responses through upregulation of IL-10 production, suppression of T cell proliferation, and inhibition of antigen presentation [38–40]. Furthermore, cystatin derived from the gastrointestinal nematode *Haemonchus contortus* (HCcyst-3) also exhibited immunosuppressive effects to inhibit MHC-II expression, phagocytosis, and inflammatory cytokine profiles [41]. In *T*. *spiralis*, the first cystatin isoform, which was identified and named multi-cystatin-like domain protein 1 (MCD-1), could not inhibit papain-like cysteine proteases [42]. *T*. *spiralis* cystatin-like protein (Ts-CLP) is the second isoform identified in the cDNA library of L1 and encodes a 45.9 kDa protein. *E*. *coli-*expressed rTs-CLP protein exhibited potentials for immunodiagnostics of trichinellosis in pig and vaccines in a mouse model [43].

In this study, a novel member of the cystatin family, which shows low sequence homology but structural conservation with type 2 cystatins, has been identified in the ES-L1 of *T*. *spiralis* [37]. Besides that, sequence analysis of TsCstN indicates a signal peptide, an N-glycosylation site, and two potential disulfide bonds, which are typical characteristics of type 2 cystatins. The multiple sequence alignment of TsCstN with homologs suggested that TsCstN did not present the conserved domains of type 2 cystatins, including glycine residue at N-terminus, QXVXG motif at L1, and PW motif at L2. The crystal structure of salivary cystatin from the soft tick, *Ornithodoros moubata*, suggested that interaction of the cystatin with papain-like cysteine peptidases is mediated by the association of N-terminus, L1, and L2 to form a wedge-like structure that interacts with the active site cleft of cysteine proteases [44]. The conserved glycine residue at the N-terminus was proposed to function as a hinge which facilitates the flexibility of the N-terminal region to adjust the conformation related to target enzyme binding [44]. At the N-terminal region of TsCstN, glycine is replaced with alanine; both of these bases are classified for exhibiting the same physical property, being hydrophobic and nonpolar, and possessing an aliphatic R group. However, site-directed mutagenesis between glycine and alanine should be performed to clarify replacing functions between them. Other two conserved motifs, QXVXG and PW, typically found in L1 and L2 of type 2 cystatins, respectively, could not be observed in TsCstN, when DXDXG and SG are substituted. Mutations at the G, QXVXG, or PW motifs of *Ascaris lumbricoides*-cystatin (Al-CPI) demonstrated that double mutations of Q50E + V52G and P97G + W98G, and combined mutations of G6E + Q50E + V52G + P97G + W98G, respectively, critically reduced inhibitory capacity of Al-CPI against CatB, CatC, CatL, and CatS [45]. Sialostatin L derived from the deer tick, *Ixodes scapularis*, exhibited substitution at the PW motif to lose the broad cysteine proteases inhibition but specifically inhibited CatL [46]. For TsCstN, substitution at the PW motif of L2 was also detected, which may have contributed to its protease specificity. Mutations that target crystallography and biochemical properties, especially site-directed mutation of TsCstN affecting protease inhibition, would be prioritized for further elucidation.

The expression of recombinant TsCstN (rTsCstN) in prokaryotic expression systems was confirmed using UV far CD spectrometry. The result indicated that the composition of secondary structure in rTsCstN (S3 Fig) is quite similar to that of the wild-type cystatin C [35]. The biochemical property of rTsCstN was confirmed by inhibiting the activity of cysteine proteases. Three lysosomal cathepsins (CatB, CatL, and CatS) were selected for evaluation owing to their significance in various physiological processes and host immune responses [47]. CatS and CatL are critically important for antigen processing by degrading MHC invariant chain (li) in antigen-presenting cells (APC) leading to the inhibition of MHC-II formation [48]. The absence of CatS, which is involved in MHC-II expression, and CatL deficiency, prevent the invariant chain (li) to CLIP in the cortical thymic epithelial cells resulting in the MHC-II surface expression stalling [49–51]. CatL is similar to CatB and is involved in the hydrolysis of extracellular matrix component and intracellular protein degradation [52]. However, the result demonstrated that rTsCstN effectively inhibited CatL, but not CatB and CatS. The unique specificity of rTsCstN to CatL may be caused by substitution at the PW motif of L2 as previously observed in the sialostatin L mentioned above [46]. The inhibitory activity of rTsCstN was dependent on pH; pH 5.0–6.0 was optimal for rTsCstN to inhibit CatL activity. In the lysosome, CatL was activated at pH 5.5–6.0, which is the pH of newly formed lysosomes [53]. Incubation of rTsCstN with mBMDMs demonstrated that rTsCstN could be internalized into lysosomes of the macrophages. Not only rTsCstN, but recombinant cystatins of other parasitic nematodes, HCcyst-3 (*Haemonchus contortus*) and AvCystatin (*Acanthocheilonema viteae*), were also taken up by host monocytes and macrophages, respectively [54, 55]. These may support the role of rTsCstN and other nematode cystatins in the direct inhibition of CatL or other cysteine proteases inside the lysosomes.

Apart from their ability to inhibit proteases, the parasite cystatins can modulate cytokine responses in macrophages. The filarial cystatins can induce the production of several cytokines. A previous study demonstrated that *O*. *volvulus* cystatin primed an early TNF-α response in human PBMCs, followed by a downregulation of the IL-12 production and a massive increase in cellular IL-10 production [39]. In mouse models, AvCystatin suppressed inflammation by the upregulation of IL-10 produced by macrophages both *in vitro* and *in vivo* [55–58]. In this study, rTsCstN-treated + LPS-stimulated mBMDMs significantly suppressed the production of pro-inflammatory cytokines, TNF-α, IL-1β, and IFN-γ, and expression of iNOS, which were produced during classical macrophage activation (M1) [59]. In contrast to filarial cystatins, rTsCstN did not induce the M2 macrophage phenotype, which could not increase regulatory cytokine and M2 marker levels [59]. As mentioned previously, TsES diminished the production of pro-inflammatory cytokines but promoted alternatively activated macrophage and regulatory cytokines productions both *in vitro* and *in vivo* [6, 7, 60]. These imply that the immunomodulatory properties of TsES may affect several molecules, including TsCstN, which need to be identified and characterized further soon.

Interference with the antigen presentation process of macrophage and the dendritic cell was observed during treatment with nematode cystatins. Nippocystatin (NbCys) derived from the ES products of *Nippostrongylus brasiliensis* suppressed processing of ovalbumin (OVA) by splenocyte lysosomal cysteine proteases of OVA-immunized mice [61]. Onchocystatin downregulated the expression of MHC-II and CD86, but did not affect the surface expression of CD40 and CD80 [39]. Furthermore, HCcyst-3 of *H*. *contortus* was able to inhibit MHC-II expression on goat monocytes in a dose-dependent manner [41]. These study results are consistent with that of our present study, which suggested that TsCstN significantly suppressed MHC-II expression on LPS-stimulated mBMDMs. However, TsCstN has no impact on the expression of costimulatory molecules, CD80 and CD86. In a molecular mechanism, nematode cystatins interfere with the formation of peptide-MHC-II complexes in macrophages and dendritic cells through the inhibition of lysosomal cysteine proteases, which are involved in two important processes, including 1) the inhibition of cysteine proteases that degrade protein to antigenic peptides before MHC-II binding and 2) cysteine protease cleavage of the MHC-II-associated invariant chain to create a CLIP peptide bond in the MHC-II groove [62]. However, the molecular mechanism of TsCstN interference with the antigen presentation process of macrophage and other APCs should be investigated promptly.

In conclusion, TsCstN is a novel cystatin derived from ES products of *T*. *spiralis* L1, which could demonstrate an immunomodulatory role in downregulating pro-inflammatory cytokine and M1 marker, iNOS, of LPS-stimulated mBMDM production. Moreover, TsCstN may interfere with an antigen presentation process of macrophage through inhibition of cathepsin L, which affects MHC-II expression. In the future, the immunomodulatory properties of TsCstN on other immune cells, the interaction between rTsCstN-primed macrophages and T cells, and the effect of TsCstN on inflammation and autoimmune mouse models should be investigated.

## Supporting information

S1 Table The gene-specific primers for qRT-PCR. (TIF)

S2 Table Top 10 threading templates used by I-TASSER. (TIF)

S3 Table Top 10 identified structural analogs in PDB. (TIF)

S1 Fig Fractionation of ES-L1 using anion exchange chromatography. M; protein ladder, lane 1 to 9; protein fraction number 1 to 9.
(TIF)

S2 Fig Analysis of the secondary structure of rTsCstN using Circular Dichroism (CD) spectroscopy. (TIF)

S3 Fig Inhibitory activity of rTsCstN against trypsin demonstrated that rTsCstN could not inhibit trypsin activity. (TIF)

S4 Fig The Z-stack analysis to confirm the internalization of rTsCstN into LPS-stimulated mBMDMs. rTsCstN was chemically tagged with FITC (green), lysosome was labeled with rabbit anti-human LAMP1 IgG coupled with Cy3-conjugated donkey anti-rabbit IgG (red), and nucleus was counterstained with Hoechst 33342 (blue). a–i, Multiple images were taken at different focal distances for variable depth.
(TIF)

S5 Fig Anti-inflammatory cytokine (IL-4, IL-10, and TGF-β) exhibited no significant difference between rTsCstN-treated + LPS-stimulated mBMDMs and LPS-stimulated-only mBMDMs (*P* > 0.05). The results are expressed as mean ± SD. The experiments were performed in triplicate with three independent experiments. One-way ANOVA followed by a Bonferroni multiple comparison test were used for analysis.
(TIF)
