# Supplementary figures and images for: A novel cystatin derived from Trichinella spiralis suppresses macrophage-mediated inflammatory responses
Source: PLoS Negl Trop Dis. 2020 Apr 1;14(4):e0008192. doi: 10.1371/journal.pntd.0008192 (PMC7153903; doi:10.1371/journal.pntd.0008192)

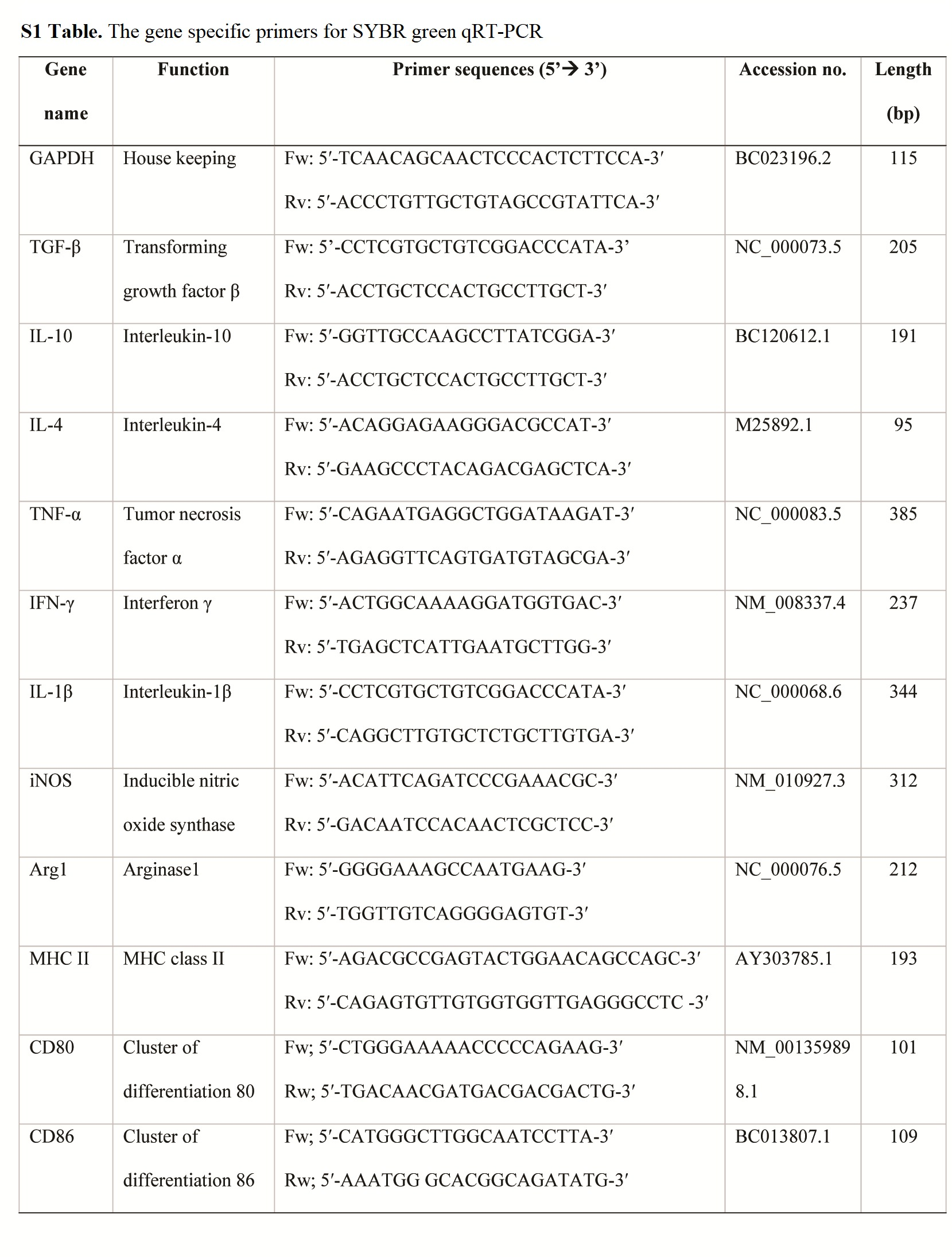

Supplement: S1 Table — (TIF) [file pntd.0008192.s001.tif]

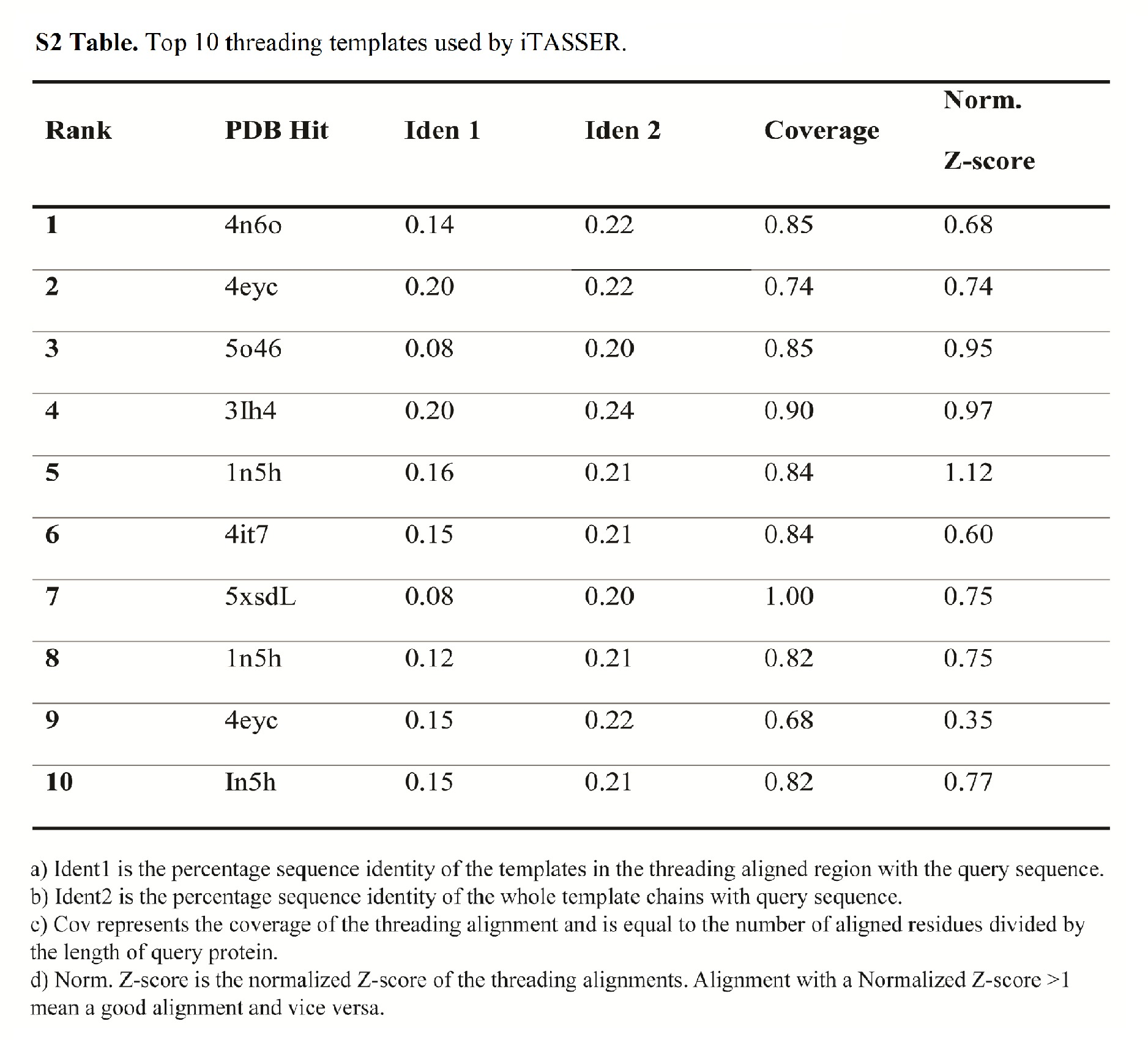

Supplement: S2 Table — (TIF) [file pntd.0008192.s002.tif]

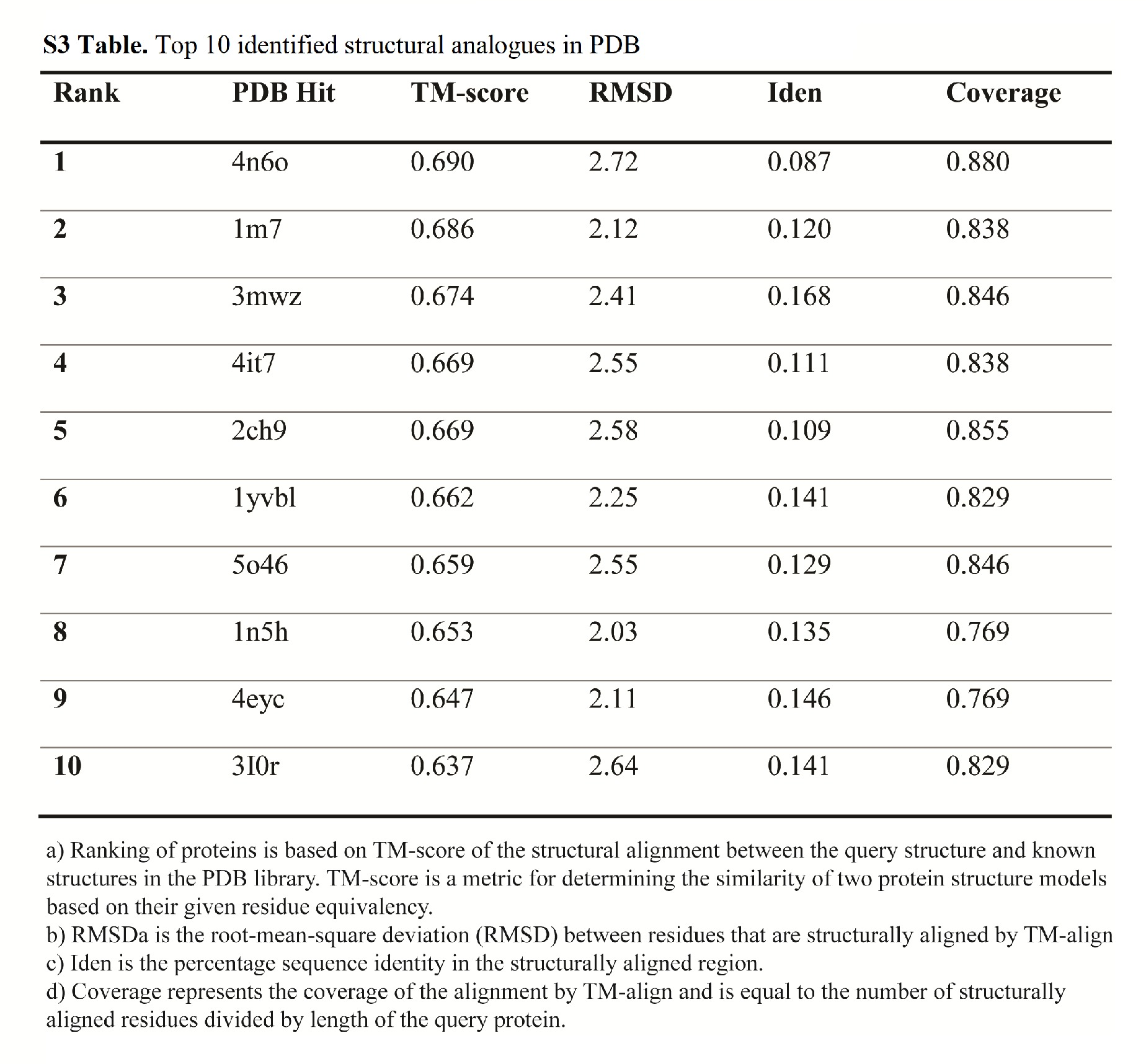

Supplement: S3 Table — (TIF) [file pntd.0008192.s003.tif]

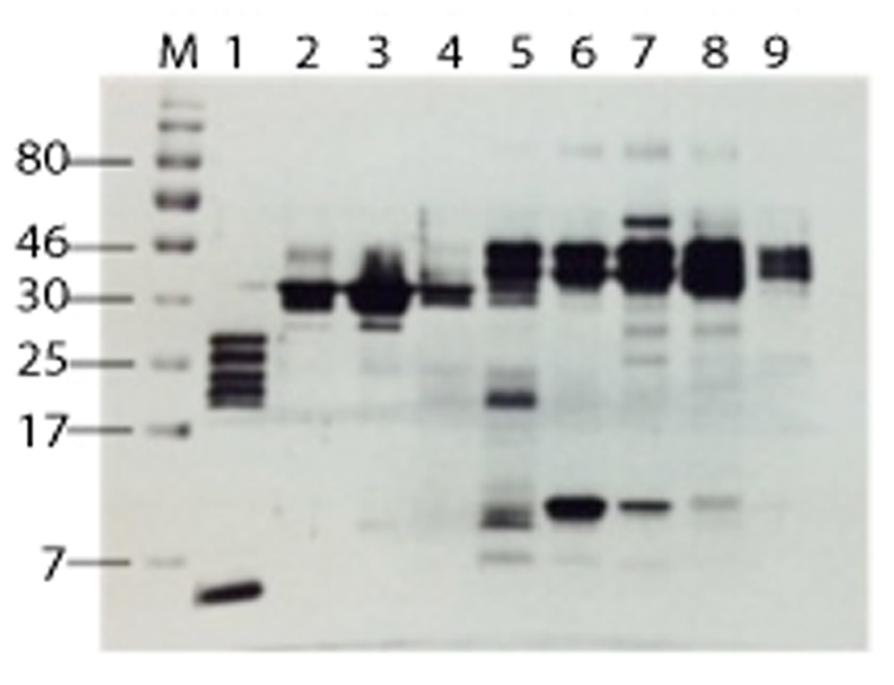

Supplement: S1 Fig — M; protein ladder, lane 1 to 9; protein fraction number 1 to 9. (TIF) [file pntd.0008192.s004.tif]

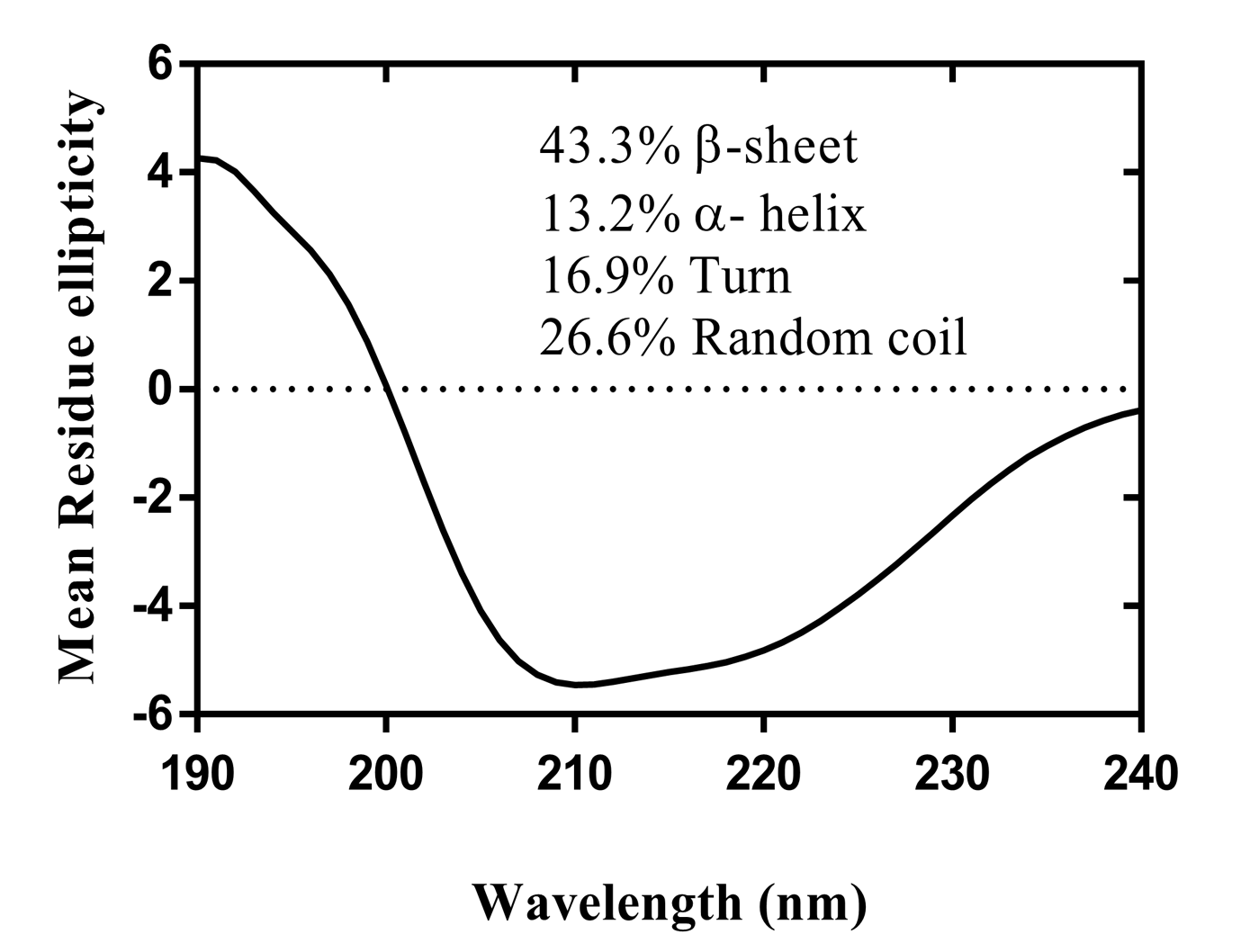

Supplement: S2 Fig — (TIF) [file pntd.0008192.s005.tif]

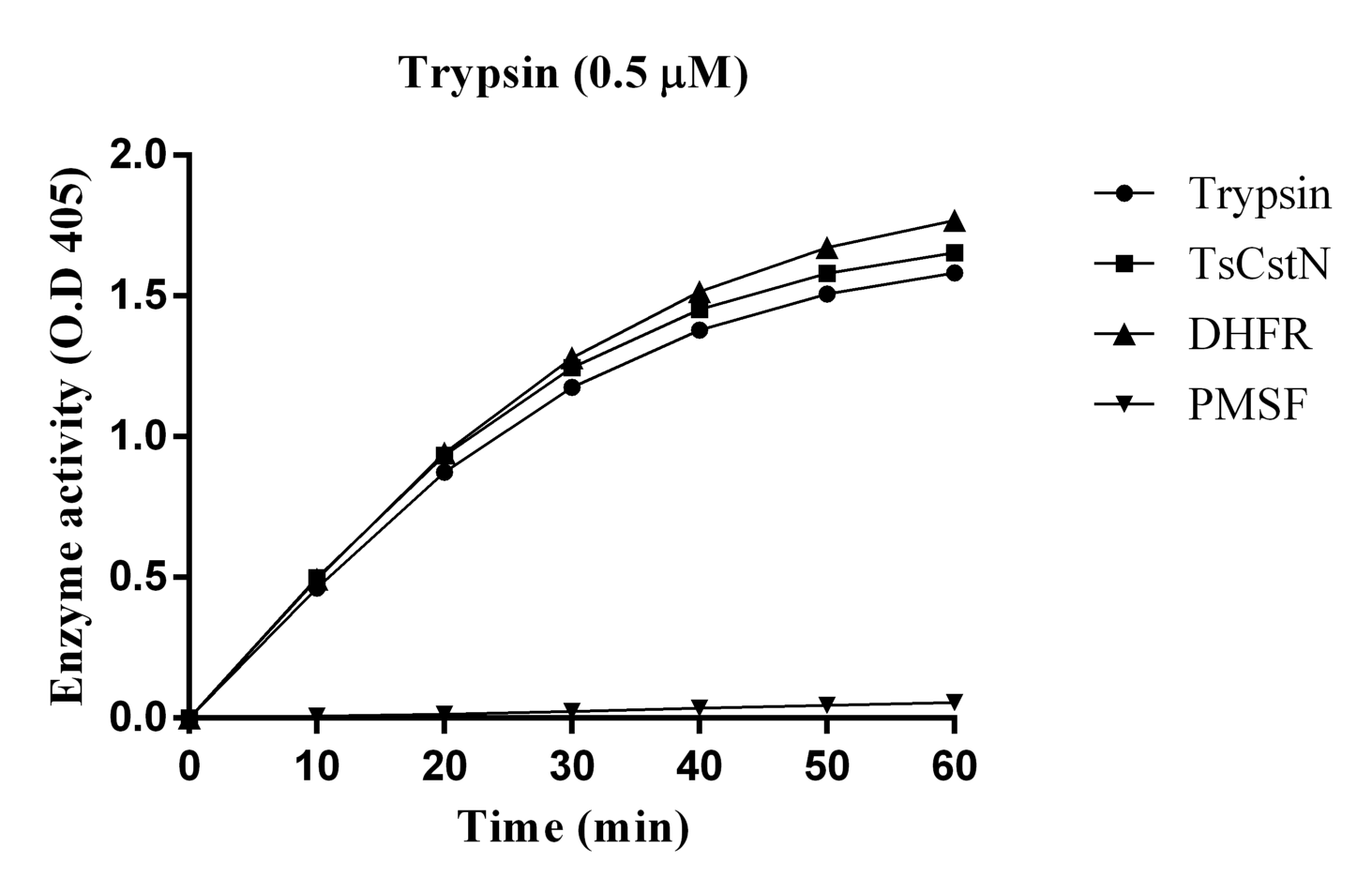

Supplement: S3 Fig — (TIF) [file pntd.0008192.s006.tif]

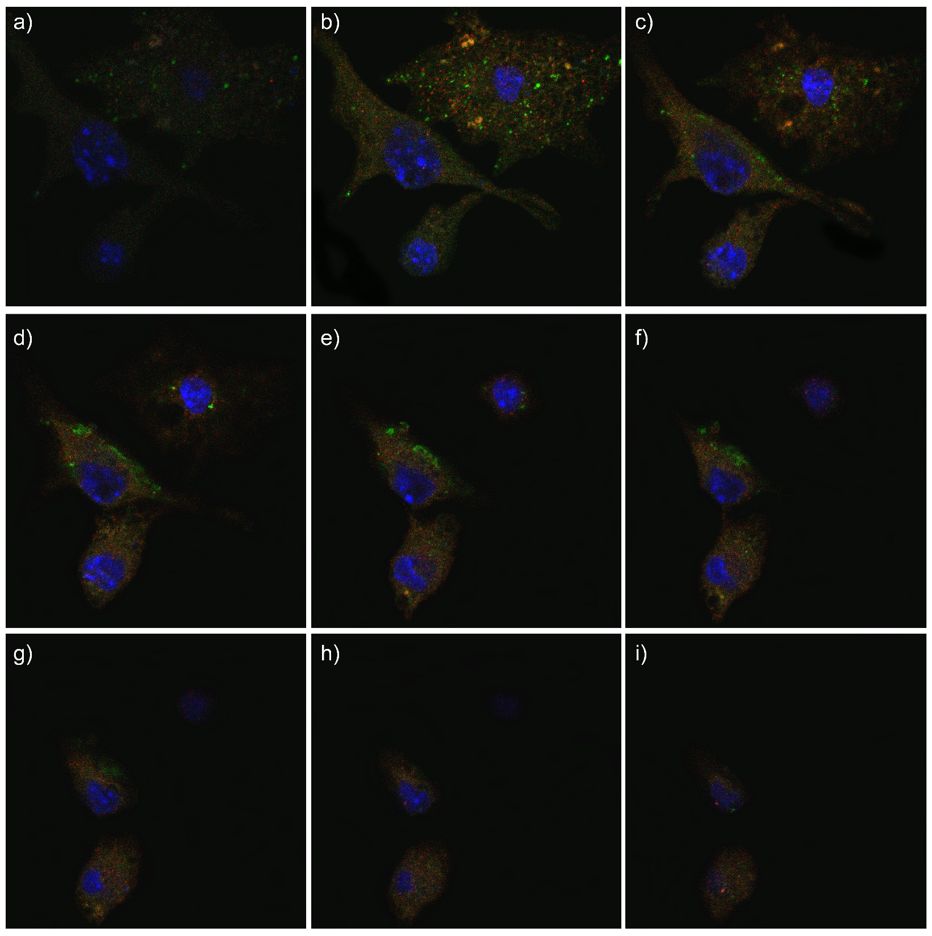

Supplement: S4 Fig — rTsCstN was chemically tagged with FITC (green), lysosome was labeled with rabbit anti-human LAMP1 IgG coupled with Cy3-conjugated donkey anti-rabbit IgG (red), and nucleus was counterstained with Hoechst 33342 (blue). a–i, Multiple images were taken at different focal distances for variable depth. (TIF) [file pntd.0008192.s007.tif]

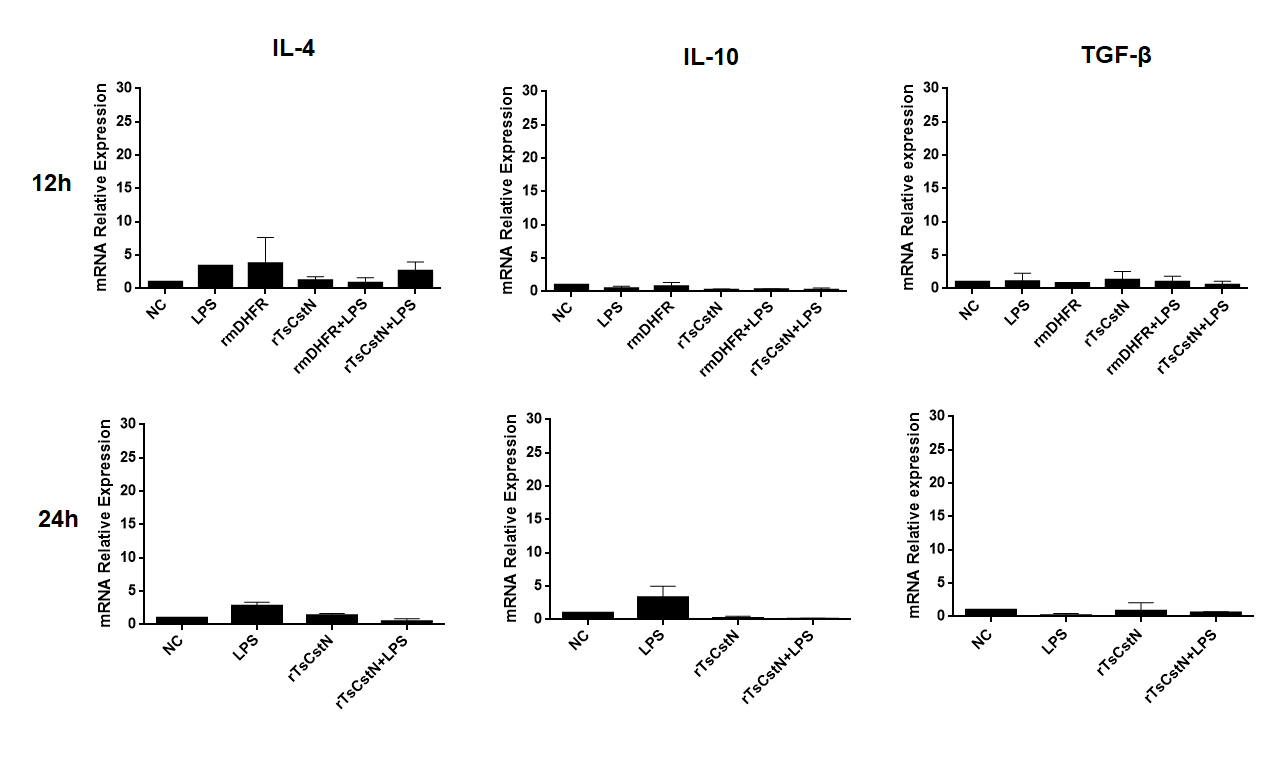

Supplement: S5 Fig — The results are expressed as mean ± SD. The experiments were performed in triplicate with three independent experiments. One-way ANOVA followed by a Bonferroni multiple comparison test were used for analysis. (TIF) [file pntd.0008192.s008.tif]
